# A Pilot Characterization of the Human Chronobiome

**DOI:** 10.1038/s41598-017-17362-6

**Published:** 2017-12-07

**Authors:** Carsten Skarke, Nicholas F. Lahens, Seth D. Rhoades, Amy Campbell, Kyle Bittinger, Aubrey Bailey, Christian Hoffmann, Randal S. Olson, Lihong Chen, Guangrui Yang, Thomas S. Price, Jason H. Moore, Frederic D. Bushman, Casey S. Greene, Gregory R. Grant, Aalim M. Weljie, Garret A. FitzGerald

**Affiliations:** 1 Department of Systems Pharmacology and Translational Therapeutics, at the University of Pennsylvania Perelman School of Medicine, Philadelphia, PA 19104 USA; 2 Department of Medicine, at the University of Pennsylvania Perelman School of Medicine, Philadelphia, PA 19104 USA; 3 Department of Microbiology, at the University of Pennsylvania Perelman School of Medicine, Philadelphia, PA 19104 USA; 4 Institute for Biomedical Informatics, at the University of Pennsylvania Perelman School of Medicine, Philadelphia, PA 19104 USA; 5 Institute for Translational Medicine and Therapeutics (ITMAT), at the University of Pennsylvania Perelman School of Medicine, Philadelphia, PA 19104 USA; 6 Department of Genetics, at the University of Pennsylvania Perelman School of Medicine, Philadelphia, PA 19104 USA; 7 Present Address: Division of Gastroenterology, Hepatology, and Nutrition, at Children’s Hospital of Philadelphia, Philadelphia, PA 19104 USA; 8 Present Address: Department of Food Science and Experimental Nutrition, Food Research Center (FoRC), School of Pharmaceutical Sciences, University of São Paulo, São Paulo, Brazil

## Abstract

Physiological function, disease expression and drug effects vary by time-of-day. Clock disruption in mice results in cardio-metabolic, immunological and neurological dysfunction; circadian misalignment using forced desynchrony increases cardiovascular risk factors in humans. Here we integrated data from remote sensors, physiological and multi-omics analyses to assess the feasibility of detecting time dependent signals - the chronobiome – despite the “noise” attributable to the behavioral differences of free-living human volunteers. The majority (62%) of sensor readouts showed time-specific variability including the expected variation in blood pressure, heart rate, and cortisol. While variance in the multi-omics is dominated by inter-individual differences, temporal patterns are evident in the metabolome (5.4% in plasma, 5.6% in saliva) and in several genera of the oral microbiome. This demonstrates, despite a small sample size and limited sampling, the feasibility of characterizing at scale the human chronobiome “in the wild”. Such reference data at scale are a prerequisite to detect and mechanistically interpret discordant data derived from patients with temporal patterns of disease expression, to develop time-specific therapeutic strategies and to refine existing treatments.

## Introduction

The molecular circadian clock coordinates our body rhythms entrainable by environmental cues, such as light, to the 24 hour solar cycle. The master clock, located in the suprachiasmatic nucleus communicates with and is influenced by molecular clocks in peripheral tissues^1^. The system is highly conserved and tightly regulated by feedback and feed forward transcriptional loops, the elements of which exhibit a high degree of genetic redundancy^2^. A robust temporal organization is achieved by the functional overlap between many of the molecular circadian clock genes; however, nuanced differences, such as differential responsiveness to photic stimuli^3^, might impact chronotypes. Studies in model systems have implicated the clock as an integrative network across tissues of particular relevance to metabolism, immune function and vascular homeostasis^4^.

In humans, many aspects of physiology, including body temperature, blood glucose, catecholamines, insulin and many hormones, including melatonin, cortisol, TSH, ghrelin, leptin and prolactin undergo diurnal variation^5^, meaning that daily patterns can be discerned. These rhythms lose amplitude and synchrony with age in both humans and mice, and deletion of core clock genes in mice has been associated with accelerated aging^6,7^. However, more recent studies have suggested that disruption “off target” effects of these transcription factors may account for some of these phenomena^8–10^. In humans, the incidence or severity of many diseases, such as asthma, myocardial infarction, stroke and depression exhibit diurnal variation^4^. Similarly, the targets of many drugs oscillate, as do enzymes and transporters relevant to drug metabolism^11^. Despite this and the long recognized time dependent variation in disposition of many commonly used drugs, there has been little exploitation of chronotherapy in clinical practice^12^.

Indeed, our understanding of the role of the molecular clock in humans is limited^13^. The use of forced desynchrony protocols has permitted segregation of clock driven circadian rhythms from diurnal variability secondary to environmental exposures. Endogenous and environmentally driven rhythms often coincide, but may be out of phase, as is the case with blood pressure, where the morning surge associated with increased cardiovascular morbidity does not temporally align with the endogenous peak in blood pressure, revealed by forced desynchrony, that occurs in the evening^14^. The clinical implications of such divergence are unknown.

Although useful, such studies are performed in highly artificial circumstances in which light and other environmental cues are carefully controlled. In recent years the development of technologies for multiscale “omics” and remote sensors afford new opportunities to explore characterization of the chronobiome of humans free ranging “in the wild”, that is, not sequestered in artificial environments.

Studies in model systems have demonstrated the role of the clock in regulation of the genome^15^, the epigenome^16^, the metabolome^17^, the proteome^18^ and the microbiome^19–21^ as well as in the oscillation of temperature^22^, activity^23^ and blood pressure^24^. However, before we can explore how dysfunction in these outputs might relate to expression of human disease, we must establish the ability to discriminate an oscillatory signal from analytical and environmental noise in healthy volunteers and determine the influence on this physiological chronobiome of such variables as gender and age. In this regard, the detectable diurnal variation in body temperature, hormones and blood pressure might serve as “internal standards” for more novel technologies.

Here, we report a pilot study designed to gather preliminary information on the variability in healthy volunteers of the diurnal oscillation of cardiovascular and behavioral phenotypes and of diverse “omics” outputs. Despite the expected intra- and inter-individual variability in behavior, a clear pattern of time dependent oscillation of blood pressure, activity, light exposure, communications and food consumption was detected. Morning- versus evening-dependent differences in both the oral and rectal microbiome abundances were clearly evident, while detection of time-of-day variation in the metabolome, proteome and transcriptome was apparent, but constrained by the number of sampling times and by sample size. We achieved a first level of data integration suggesting multidimensional fingerprints unique to each person.

## Methods

We enrolled 6 healthy male volunteers (32.3 ± 3.6 years of age, BMI 25.2 ± 3.4 kg/m^2^) after approval by the Institutional Review Board of the University of Pennsylvania (Federalwide Assurance FWA00004028; IRB Registration: IORG0000029) that included an institutional security and privacy information impact assessment and registration (clinicaltrials.gov NCT02249793). Informed consent was obtained from all subjects. This clinical research study was carried out in accordance with relevant guidelines and regulations. The main exclusion criteria consisted of travel across time zones and irregular work hours, e.g. shift work. Volunteers were studied over 4 months to collect data on activity, sleep patterns, light exposure and communication, as well as being deeply phenotyped during two 48 hour periods, 2 weeks apart. Biospecimens (plasma, serum, saliva, oral and rectal swabs) were collected from these outpatients in the Center for Human Phenomic Science (CHPS, University of Pennsylvania) at 12 hourly intervals, thus generating a time series of 5 consecutive sample collections during one single 48 hour session, i.e. 0 hrs = morning, 12 hrs = evening, 24 hrs = morning, 36 hrs = evening, and 48 hrs = morning. Plasma and saliva metabolites were analyzed using LC-MS as previously described^25^, a pre-specified protein panel was run on the SomaLogic platform^26^, the microbiomic analysis in saliva and from buccal and rectal swabs was conducted as established earlier^27,28^, and in-house qPCR was used for expression analysis of a small, selected panel of genes.

A triaxial actigraph device (wActiSleep-BT) recorded accelerometer and light sensor data with subsequent wear time analysis and sleep scoring in ActiLife 6.0 software. This achieved quantitative outputs for steps, energy expenditure and metabolic rate as additional outputs in this domain. The HIPAA-compliant Ginger.io platform^®^, consisting of an android mobile phone application and a web dashboard, was used to monitor cell phone calls and SMS messaging activity in real time. The application gathered communication and mobility data through a background process and transmitted encrypted data to firewall protected linux-based servers with access control lists. Blood pressure monitoring was performed in the ambulatory subjects using clinically validated devices (Spacelab 90207). Intake of food and beverages was collected with the SmartIntake^©^ smartphone application, a validated remote food photography method^®^^29,30^. To facilitate data quality and completeness, the app included Ecological Momentary Assessment (EMA) methodology to remind participants to capture images of the foods and beverages that they consumed. These reminders were text messages that were scheduled for delivery at the personalized meal times of the participants. The responses to EMAs were tracked in near real-time, which allowed us to identify quickly if data collection problems occurred. The app sends participants food/beverage images and accompanying food identifier data (e.g., barcodes, PLU numbers, food descriptions) to a server located at the Pennington Biomedical Research Center where bionutritionists analyze the images to estimate food/beverage intake based on the Food Photography Application© program. This allows the operator to identify a match for each food from the Food and Nutrient Database for Dietary Studies 5.0 and other sources, such as manufacturer’s information and Nutrition Fact Panels, to calculate energy and nutrient intake.

For the bioinformatics analysis, we adopted several packages in R and CircOS for data management, integration and visualization. The web-based version control repository GitHub was used as code development platform (https://github.com/itmat/chronobiome/). In addition to standard descriptive statistics, we applied permutation tests, principal component analysis, principal coordinate analysis, circadian multiresolution analyses, cosinor method, Ingenuity pathway analysis, variance correlation analysis, and a time-versus-subject contribution to variance analysis.

## Results

**Figure 1:**
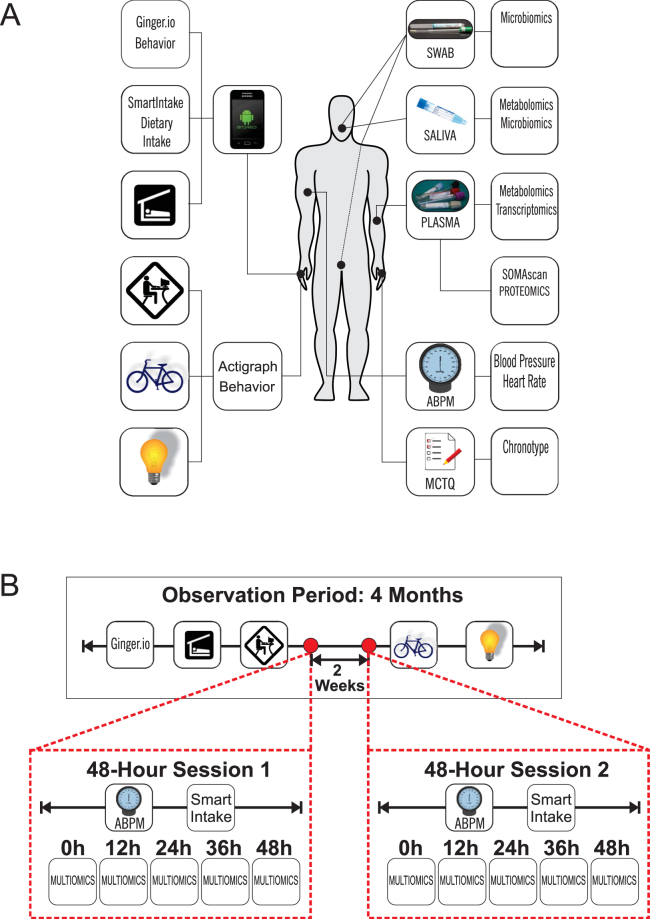
Study Design. (**A**) Study participants were equipped with remote sensing devices to collect behavioral and environmental data including activity, communication, mobility, sleep-wake times, dietary intake and light exposure. Clinical assessments included ambulatory blood pressure and heart rate. (**B**) The observation time for the biosensor-derived data was a total of four months with two 48-hour sessions (Session 1 & 2) scheduled two weeks apart to extend the biosensor platform by ambulatory blood pressure monitoring (ABPM) and timestamped dietary intake (SmartIntake) as well as by collection of timed biospecimens for multiomics analysis at 12-hour intervals.

We successfully integrated and analyzed this multidimensional dataset, roughly 2.2 million data points collected from 6 healthy volunteers over the course of 4 months, including two 48-hour sessions of additional deep phenotyping (Fig. 1, Figure S1). This led to several insights relevant to the pursuit of future studies in the field of human chronobiology.

**Figure 2:**
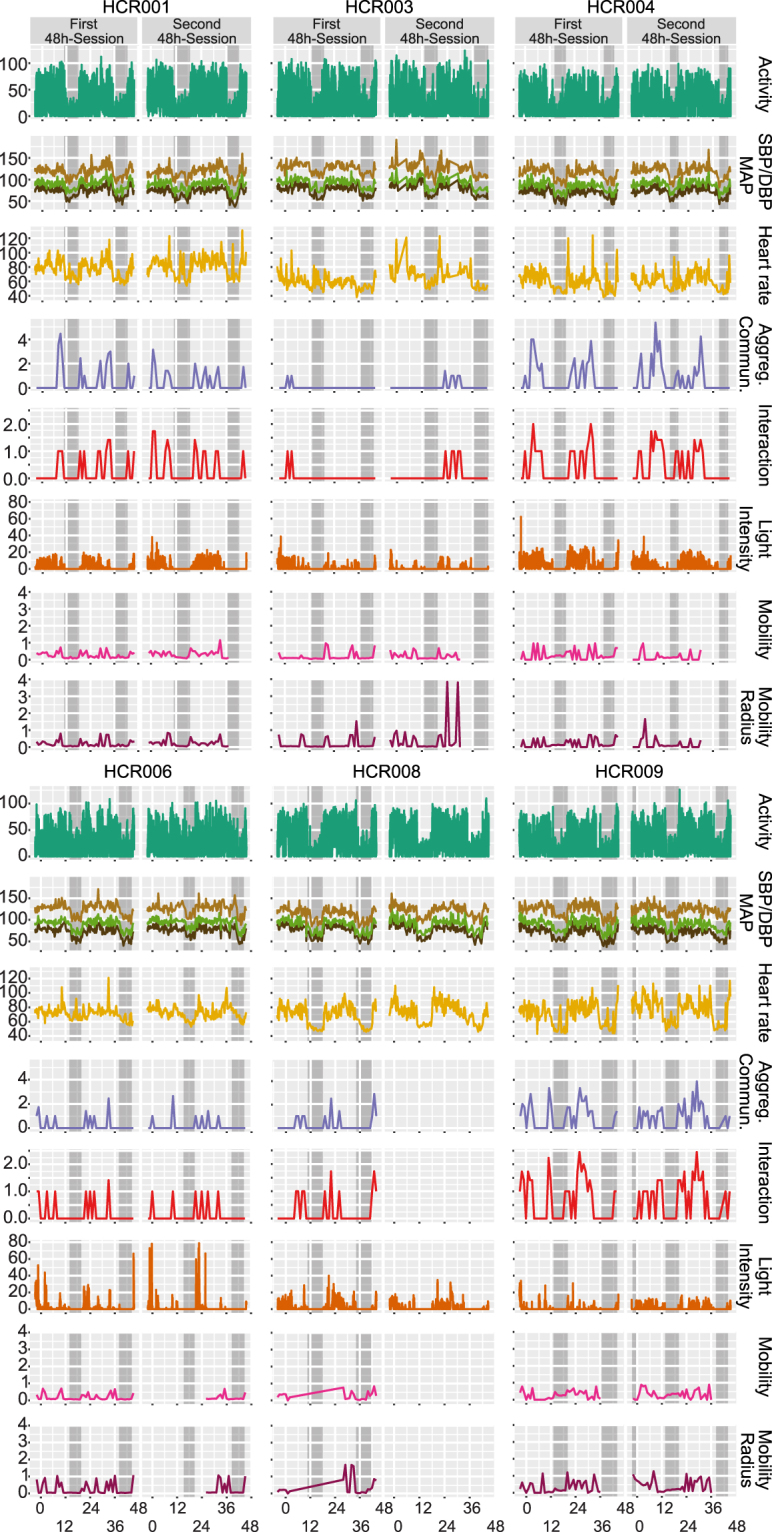
Remote Sensing, Blood Pressure & Heart Rate. Horizontal panels display the following data for each of the n = 6 participants: activity [square root of vector magnitude], systolic, mean arterial, and diastolic blood pressure [mmHg SBP and DBP], heart rate [bpm], aggregate communication [square root of the sum of counts of phone calls and text messages], interaction [square root of counts ∙ min^−1^], light intensity [square root of lux ∙ min^−1^], and mobility/mobility radius [square root of miles] sampled over 48 hours during the first and second sessions. Self-reported sleep times are marked as grey boxes.
ii. Locomotor activity was highest during self-reported wake times, on average 1904 counts ∙ min^−1^ using the raw data outputs of the Actigraph’s accelerometer as reference. This compared to just 307 counts ∙ min^−1^ during self-reported sleep times (Fig. 2, Figure S2).
iii. Remote sensors indicated that aggregate communication happened during self-reported wake times (18.7 calls and sms/wake hours) with close to none at night (0.9 calls and sms/sleep hours). The GPS informed readout of mobility confirmed the urban setting of our study. Participants traveled on average 1.6 miles/wake hours with absent mobility during self-reported sleep (0.2 miles/sleep hours) (Fig. 2, Figure S2).
iv. Ambient light intensities followed the patterns of self-reported wake/sleep times where the wrist-worn luxmeter detected light on average 42.3 lux ∙ min^−1^ during wake hours compared to 2.7 lux ∙ min^−1^ during sleep hours (Fig. 2, Figure S2).
v. Food intake did not occur during self-reported sleep times (Fig. 3, Figure S3).

First, we sought to assess the validity of our dataset. We see this accomplished on several levels:

i. A clear diurnal signal, as expected in these healthy young urban professional males, was detectable in blood pressure, dipping at night on average by 19.4 ± 3.2 mmHg in mean arterial pressure. This was accompanied by a nocturnal drop in heart rate by an average of 16.5 ± 6.6 bpm (Fig. 2).

**Figure 3:**
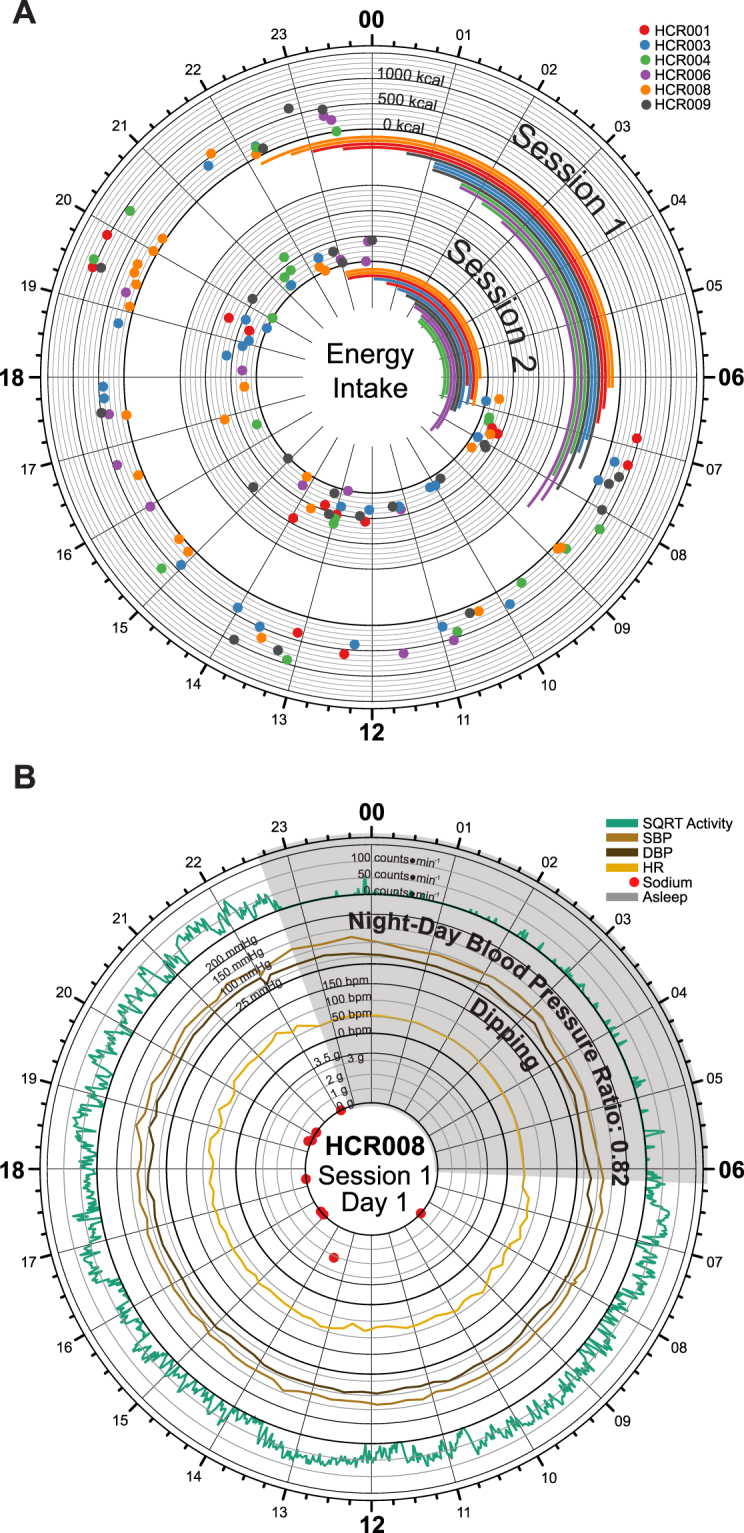
Dietary Intake by Remote Food Photography. (**A**) Time-of-day dependent energy intake for all subjects during session 1 (outer circle) and session 2 (inner circle). The data in each session track display energy intake for two full days of each session. 24-hour clock times are listed around the edge of the plot, with “00” corresponding to midnight, and “12” corresponding to noon. Dots are color-coded by subject and indicate the energy intake (kcal) at the corresponding clock time. Dark axis lines mark 0, 500, 1000, and 1500 kcal consumed. Lighter axis lines mark energy intake in 100 kcal steps. Sleep spans are also color-coded by subject and are indicated using the bars below each of the corresponding session. (**B**) Time-of-day dependent fluctuations in activity (counts * min^−1^, green), systolic (mmHg, brown) and diastolic (mmHg, black) blood pressure, heart rate (bpm, orange) plotted with time-specific dietary intake of sodium (g, red); sleep time marked as grey wedge. As expected, a dipping phenotype in blood pressure was observed for this subject.
vi. Plasma cortisol showed the expected time-of-day-dependent variance with relative levels of 1.3 ± 0.4 higher in the morning than 0.8 ± 0.2 in the evening. Though noisier, cortisol levels in saliva followed this pattern (Fig. 4).

**Figure 4:**
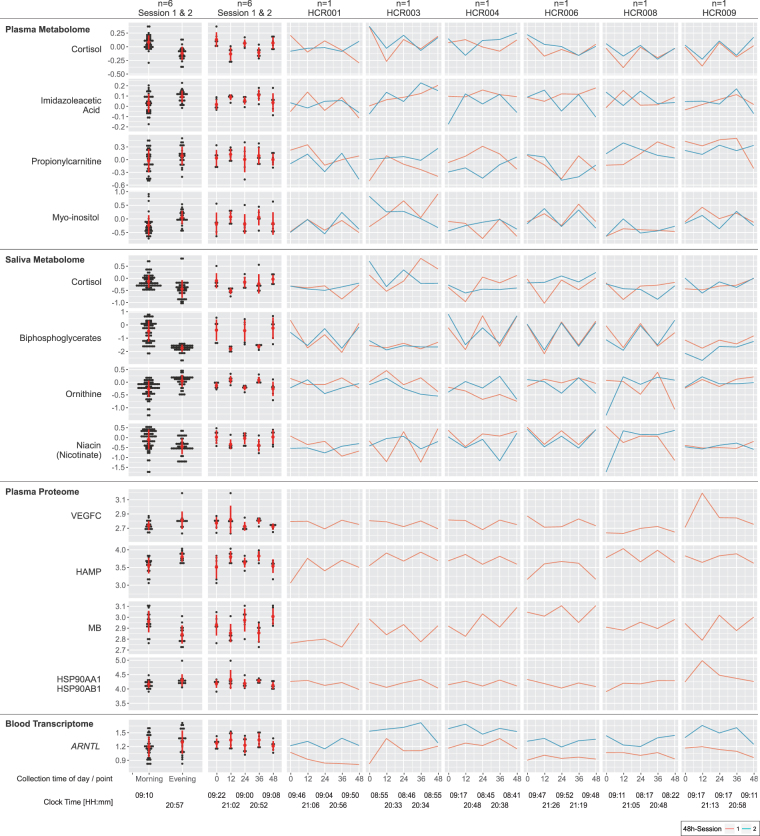
Metabolomics, Proteomics & Transcriptomics. Time-of-day dependent differences in metabolite/protein/gene levels are displayed selecting the top-ranked candidates per non-parametric statistical test: (i) aggregated by morning/evening for all n = 6 subjects (left column), (ii) aggregated by time point (0 h - morning, 12 h - evening, 24 h - morning, 36 h - evening, 48 h - morning) for all n = 6 subjects (second left column), and (iii) individual time series from session 1 (red) and session 2 (blue) for each subject (6 columns to the right). The red circles and bars in the two left-most columns indicate the mean and standard deviations for each aggregated dataset, respectively. Please note that data were visualized on a log10 scale.

In summary, we were able to detect internally consistent, time dependent patterns in blood pressure, heart rate, cortisol, activity, communication, mobility and light consistent with the physiological and behavioral expectations for this cohort in the natural setting under the conditions of this experiment.

**Figure 5:**
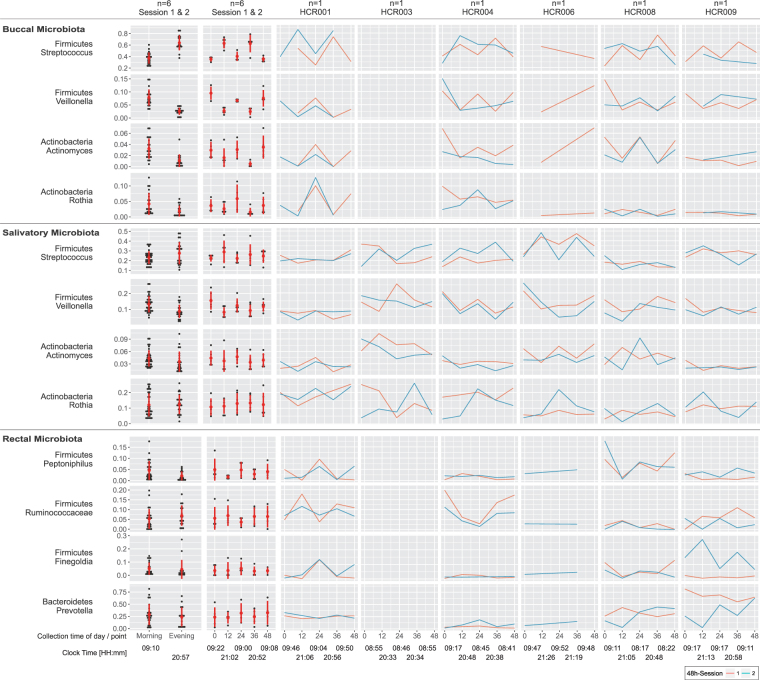
Microbiomics, Salivatory, Buccal & Rectal. Time-of-day dependent differences in the relative fraction of bacterial genera are displayed: (i) aggregated by morning/evening for all n = 6 subjects (left column), (ii) aggregated by time point (0 h - morning, 12 h - evening, 24 h - morning, 36 h - evening, 48 h - morning) for all n = 6 subjects (second left column), and (iii) individual time series from session 1 (red) and session 2 (blue) for each subject (6 columns to the right). The red circles and bars in the two left-most columns indicate the mean and standard deviations for each aggregated dataset, respectively. Please note that data were visualized on a log10 scale.

**Figure 6:**
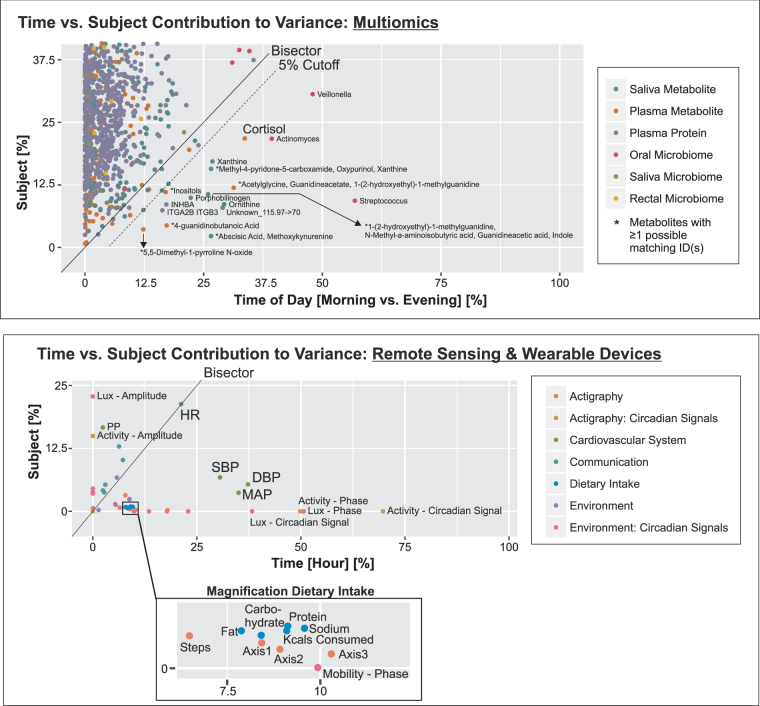
Time-versus-Subject Contribution to Variance Analysis. (**a**) Percent contribution to variance by subject versus time-of-day is displayed for the multiomic, e.g. for cortisol, as expected, the time variance contribution is higher than by subject. Note that time-of-day refers to the three morning and two evening replicates within one 48 hour session. We defined a 5% cutoff (dotted line) to discern variables with a higher time-of-day from subject contribution to variance. (**b**) Readouts collected from remote sensors and wearable devices segregate according to the percent degree of how much variance is contributed by subject versus time, e.g. for blood pressure (SBP, DBP) the variance contribution by time is higher than by subject, thus underscoring the diurnality of this phenotype. (SBP/DBP: systolic/diastolic blood pressure; MAP: mean arterial pressure; HR: heart rate; PP: pulse pressure). (Insert) This blow up magnifies for dietary food the percent contribution to variance by subject versus time-of-day. Time-specific data was collected by the phone application SmartIntake© during the 48 hrs sessions. (Axis 1–3 refer to the Actigraph’s accelerometers).

Next, we were interested to assess the comparative contributions of time (the signal) and inter-subject behavioral differences (the noise) to variability in our datasets. There are many sources of variability in these data. By partitioning the total variability, we were able to attribute how much variability was explained by time, the variable of interest in our study (see Supplemental Methods for full details). For the multiomics dataset, the permutation distribution (obtained by permuting the time points in all possible ways) revealed patterns which displayed statistically significant temporal variation between morning (0 h, 24 h, 48 h) and evening (12 h, 36 h) measurements (permutation *p*-values: plasma metabolome *p* = 0.009; saliva metabolome *p* = 0.009, saliva microbiome *p* = 0.009). We determined that 5.4% (9/166) of the plasma metabolites, 5.6% (14/250) of the saliva metabolites, 0.5% (6/1141) of the serum proteins, and 3 of the 12 most abundant genera in the oral microbiota underwent time-specific variability (Figs 4, 5 and 6a). For the metabolites, examples include cortisol in plasma, and ornithine, xanthine and porphobilinogen in saliva. The plasma proteome overall failed to attain significance (*p* = 0.56). Accordingly, variance in protein abundances was driven by inter-subject differences in the majority of cases (99.2%), whereas for some proteins variance was exclusively contributed by inter-subject differences. For the oral microbiota, three genera, Streptococcus, Veillonella, and Actinomyces revealed a predominant time-dependent variance (Figs 5 and 6a). Thus, time-of-day-dependent patterning was detected in the metabolome and the microbiome despite the paucity of sampling times and the behavioral diversity of a small number of free ranging humans. This integrative approach allows us to discern candidate oscillatory variables despite the anticipated inter-individual differences, small sample size and sparse sampling. As expected for cortisol (Fig. 6a), time (33.6%) contributes more variance than inter-subject differences (21.8%), thus demonstrating first proof-of-concept.

In our dataset obtained from remote sensors and wearables, we identified 62% of the variables to show time-specific variability. Here, variables from almost all domains, behavior (activity, mobility), cardiovascular (SDB, DBP, MAP), and environment (light), are represented (Fig. 6a). This includes several food categories - intake of energy, protein, carbohydrates, fat and sodium - where time more than inter-subject differences contributes to variability of intake, despite the unrestricted access to food under the conditions of this study (Fig. 6b bottom insert). By contrast, all readouts for communication depart from this pattern with variance reflecting inter-subject behavior more than time of day. Heart rate has equal time- and inter-subject variance, while, despite the dominant contribution of time to SBP and DBP, variance in pulse pressure mainly reflects inter-subject differences (Fig. 6b).

To parse the relative contributions of time and inter-subject differences to variability in the datasets, we conducted a principal component analysis (Figure S4). As expected, inter-subject differences explain most of the variability observed in the data clustering. Further attribution of variability was not feasible due to our ‘unsupervised’ approach. In an effort to explore which disease categories might be subject to time dependent oscillations, we performed a time-specific pathway analysis of the metabolome and the proteome. Interestingly, the statistically significant categories centered on cancer and inflammation (Figure S5), two conditions subject to clock dependent regulation in mice.

**Figure 7:**
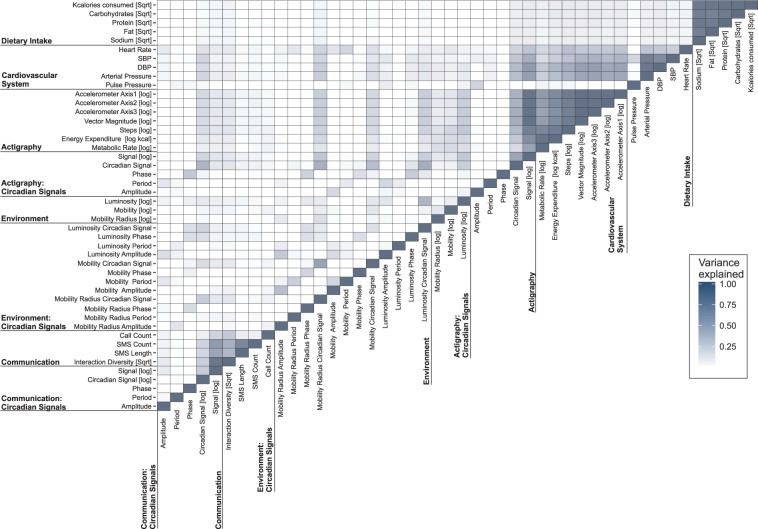
Variance Correlation Matrix. The heatmap displays the degree of variance explained across outputs collected from n = 6 healthy volunteers during the two 48-hour sessions. The percentage of variance explained (R^2^) is depicted by the color scale ranging from white, i.e. regression provides a poor fit for the indicated pair of variables, to dark blue, where the regression produces a good fit between the two variables.

To seek redundancy amongst the parameters tracked by the remote sensors and wearables, we constructed variance correlation matrices where the goodness-of-fit *p*-values produced from the linear regression analysis informed statistical significance (Fig. 7). This approach visualizes the proportion of variance observed for each variable explained by the variance observed for each other variable. For example, the outputs from the Actigraph’s accelerometers, that is axes 1, 2, and 3 to measure acceleration in three directions, correlate highly with each other as expected (e.g. R^2^ = 0.96, *Bonferroni corrected p = *1.8 * 10^−277^ between axis 1 and 2). Clusters of high correlation are evident both within-domain (e.g. between SBP and DBP [R^2^ = 72.5, *Bonferroni corrected p* = 3.2 * 10^−112^] or between heart rate and SBP [R^2^ = 23, *Bonferroni corrected p* = 2 * 10^−21^]) and between-domains, as evident between readouts of activity and cardiovascular function. Interestingly, considerable between-subject differences in the variance correlation matrices were noticeable. For example, the relationship between SBP and the oscillatory signal from activity ranged from R^2^ of 0.22 to 0.67 amongst the 6 volunteers.

## Discussion

Forced desynchrony protocols have provided valuable information on the role of the molecular clock in humans, permitting segregation of rhythmic activities driven by endogenous and environmental factors^14,31–34^. For example, disruption of endogenous rhythms results in disturbance of cardiovascular homeostasis, including a rise in blood pressure^14^. An open question is whether it is also possible to interrogate the contribution of discordant clock driven rhythmicity to time dependent expression of disease phenotypes in unrestricted settings, where the “noise” consequent to divergent behavior, therapies and concordant disease might obscure the detection of oscillatory signals of potential mechanistic relevance. A first step towards addressing this question is to perform a pilot study to determine whether oscillatory signals can even be detected in apparently healthy humans, selected for demographic homogeneity, but allowed to free range without environmental restriction. In the present study, we provide the first evidence that many such signals are detectable despite variance, even in a small number of individuals sampled infrequently, illustrating the feasibility of characterizing the chronobiome – the collective of rhythmic phenomena – of humans living “in the wild”.

Here we report the integration of multidimensional data collected via remote sensing, cardiovascular assessments and “omics” analyses. As anticipated, we see a diversity of behavioral patterns in this apparently homogenous population, purposefully standardized for age, gender and health status to increase the likelihood of detecting time dependent variations. Despite this, the small sample size and protocol violations, we see that several clock-determined diurnal readouts, i.e. blood pressure and cortisol, were internally consistent with time-dependent patterns in the volunteers’ physical activity, mobility, communication and environmental cues (ambient light exposure). While the majority of remote sensor readouts showed time-specific variability (62%), we find that inter-subject differences mainly drove variability in communication. This latter observation overlaps with findings from e-mail communications in large university-based cohorts sampled in Europe and the US^35^. This study described two broad e-mail phenotypes, one restricting use to work hours, the other persistently active during wake hours. As residents in Western societies move increasingly outside the environmental light-dark cycle, our approach might afford new avenues to investigate the health implications of this cultural change. For example, one might parse for synchrony versus asynchrony between outcome variables using circadian phase.

In our cohort, as expected, we observed high correlations between circadian phases of activity, communication, mobility and light, thus suggesting high synchronicity. Notably, time-specific phase shifts can be induced by food intake, for example, carbohydrates in the morning (compared to evening) phase-advance heart rate by three-quarters of an hour^36^.

Circadian amplitude offers yet another perspective on circadian organization. Healthy volunteers under conditions of forced desychrony variably respond with a reduction in amplitude across clock-determined oscillatory endpoints^37^; however, the relationship to adverse health effects is less clear. In our cohort, as we would expect, circadian amplitudes of different outputs correlated highly. For example, that between activity and mobility (R^2^ of 16.4%, *Bonferroni corrected-p* = 4.2 * 10^−14^), was similar to the correlation between activity and communication (R^2^ of 16.4%, *Bonferroni corrected-p* = 3.1 * 10^−14^). If and how these relationships change under acute and chronic exposure to stress remains to be seen.

Temporal patterns are discernable in the “omics” data, most pronounced in the metabolome (5.4% in plasma and 5.6% in saliva), and evident in several genera of the oral microbiome. In the case of the plasma proteome and whole blood transcriptome, more frequent analyses in larger cohorts will be necessary comprehensively to discern signal from noise. Our exploratory pathway analysis revealed that metabolomic as well as proteomic pathways associated with cancer and inflammation were enriched in a temporal fashion. Prominent interplay with circadian clocks has been described in mice for both diseases^4,38^.

These data provide a reference set for the design of larger studies comprehensively to interrogate the chronobiome. For example, we wish to determine how age and gender, two factors that interact with clock-derived outputs in model systems^21,39^, and seasonal variation^40^ modulate the human chronobiome. More detailed phenotyping will include additional analytical platforms, for example the breath metabolome^41^, and characterization of the response to time dependent metabolic^42^, inflammatory^43^ and cardiovascular^44^ perturbations of the chronobiome. Such deep phenotypic characterization will provide a comparator for investigation of chronobiomic divergence of potential mechanistic and therapeutic value in syndromes of time dependent disease expression, such as non-dipping hypertension, nocturnal asthma and depression.

## Electronic supplementary material

Supplements
