# Supplementary material for: A Pilot Characterization of the Human Chronobiome
Source: Sci Rep. 2017 Dec 7;7:17141. doi: 10.1038/s41598-017-17362-6 (PMC5719427; doi:10.1038/s41598-017-17362-6)
Supplement: Supplementary file 1 — Supplements [file 41598_2017_17362_MOESM1_ESM.pdf]

A Pilot Characterization of the Human Chronobiome.

Supplemental Materials

Carsten Skarke[1,2,5], Nicholas Lahens[1,&], Seth Rhoades[1,&], Amy Campbell[1], Kyle Bittinger[3], Aubrey Bailey[3], Christian Hoffmann[3], Randal S. Olson[4], Lihong Chen[1], Guangrui Yang[1], Thomas S. Price[1], Jason H. Moore[4,5], Frederic D. Bushman[3,5], Casey S. Greene[1,5], Gregory R. Grant[5,6], Aalim M. Weljie[1,5] & Garret A. FitzGerald[1,2,5]

[1] Department of Systems Pharmacology and Translational Therapeutics,

[2] Department of Medicine,

[3] Department of Microbiology,

[4] Institute for Biomedical Informatics,

[5] Institute for Translational Medicine and Therapeutics (ITMAT),

[6] Department of Genetics,

at the University of Pennsylvania Perelman School of Medicine, Philadelphia, PA 19104, USA

[&] These co-authors contributed equally.

Authors for correspondence:

Carsten Skarke, M.D. (cskarke@pennmedicine.upenn.edu) and Garret A. FitzGerald, M.D. (garret@pennmedicine.upenn.edu)

Supplemental Methods

Human Studies

The design of this pilot study (Figure 1) combined longitudinal assessments over four months (activity, light exposure, sleep times, and communication) with additional deep phenotyping over 48 hours (ambulatory blood pressure monitoring, biospecimen collections for multiomics biology, and food intake). The 48 hour assessments were conducted twice, two weeks apart, timed at around the midpoint of the four-month data collection. Given that acute alcohol ingestion dampens biological rhythms in humans [1,2], volunteers were asked to abstain from alcohol for both 48-hour sessions beginning 24 hours beforehand. Caffeine and high fat foods were permissible. Healthy, male volunteers were enrolled after protocol approval by the Institutional Review Board which included an institutional security and privacy information impact assessment (SPIA) and registration (clinicaltrials.gov NCT02249793). All study procedures and associated risks were explained verbally and in writing prior to obtaining consent from the study volunteers. They were non-smokers and abstained from the use of vitamins, non-steroidal anti-inflammatory drugs (NSAIDs including aspirin) and illicit drugs as assessed by cotinine (Craig Medical, Vista, CA), platelet aggregometry [3] and a urine drug screen (RDI, Poteau, OK), respectively, for at least two weeks before enrollment and at the start of each 48 hour session. After enrollment and authorization (WeP Project Agreement), the Munich ChronoType Questionnaire (MCTQ) was administered online [4]. Health status and safety was assessed by routine medical history, physical exam, and laboratory work (hematology, biochemistry, and urinalysis) at the time of screening and again on completion of the study. Vital signs were recorded and queries for adverse events were made at each clinical visit. Aside from general health, the main inclusion criterion was to own a smartphone running an Android operating system, version 2.3 or higher. The main exclusion criteria comprised travel across time zones and irregular work hours, e.g. night shifts. To assess the function of the renin–angiotensin–aldosterone system (RAAS) and kidney, biospecimens were taken twice, once during each 48 hour session, to measure urinary electrolytes and aldosterone, cortisol, renin activity, creatinine clearance, GFR, and BUN.

Biospecimens

Plasma, serum, saliva, oral and rectal swabs were collected at 0, 12, 24, 36, and 48 hours in each session. Biospecimens were stored at -80°C until analysis.

Ambulatory Blood Pressure Monitoring

The blood pressure cuff for standard adults (24 to 32 cm) was placed on the non-dominant arm. Blood pressure was measured every 15 minutes in the daytime (0600h-2200h) and every 30 minutes during the nighttime (2200h-0600h) continuously over each of the two 48 hour sessions using the Spacelabs Model 90207 (Spacelabs Medical, Issequah, WA) [5,6]. Raw data were exported in TXR file format from the Spacelab 92506 ABP Report Management System. Subject paper diaries collected self-reported awake and asleep times.

Actigraphy

A triaxial actigraph device (wActiSleep-BT, ActiGraph, Pensacola, FL) was placed on the non-dominant wrist of the subject. Subjects were advised to wear the actigraph continuously for the duration of four months. Devices were initialized with start date and time using atomic server time, no pre-defined stop date/time, at 60 Hz sampling rate for the three accelerometer axes, enabled for delay modus, steps, lux, inclinometer, and sleep while active. Raw data were exported in AGD and GT3X file format in one second epochs using ActiLife software (version 6, ActiGraph, Pensacola, FL). Each subject's AGD files were merged and reintegrated for analysis at epochs of 60 seconds. The raw data output is count [7] for each accelerometer, which is transformed into vector magnitude as a composite measure according to the $\sqrt{V^2 + H^2 + P^2}$ where *V* is the vertical axis count (axis 1), *H* is the horizontal axis count (axis 2) and *P* is the perpendicular axis count (axis 3). These data are reported, based on the 60 second epochs, in units of [counts • min^{-1}].

The proprietary actigraph software (ActiLife 6.0) was used to calculate device wear time (Actigraphy Wear Time Validation) with the Troiano technique [8] developed for detection of non-wear periods from the representative 2003-2004 NHANES dataset.

Both ActiLife (v6.0) built-in algorithms Cole-Kripke [9] and Sadeh [10] were used for sleep scoring, which labels individual epochs as either sleep or non-sleep. Here, manual corrections of sleep period identification were not conducted. In the subsequent step, the "Detect Sleep Periods" feature, based on the Tudor-Locke auto sleep period detection algorithm [11], calculated bedtimes defined as Time in Bed (TIB) and Time Out of Bed (TOB).

The Actigraph data of our study participants were compared against the NHANES cohort for counts, energy expenditure, gender, age, weight, height and BMI using the NHANES module in the ActiLife (v6.0) software.

Light Sensor

The actigraph (wActiSleep-BT, ActiGraph, Pensacola, FL) is equipped with a light sensor. Ambient light intensities were recorded in lux at 60 Hz frequency over the four months wear time. Raw data were reintegrated at 60 seconds epochs by the ActiLife software (version 6, ActiGraph, Pensacola, FL) and reported in units of [lux • min^{-1}] for this study. Average light intensities were calculated for each subject using the sleep survey to annotate each data point as 'awake' or 'sleeping'. Available data collected during the 4 month observational period, integrated at 1 minute epochs, were then averaged within each subject for the conditions 'awake' and 'sleeping'.

Microbiomics

For the separate pilot study, stool samples were collected as a pilot during an interventional study in healthy volunteers (clinicaltrials.gov registration number: NCT00682318) studying i) the interaction between marine lipids and ethanol on lipid peroxidation, and ii) the formation of putative pro-resolving lipid mediators [12,13]. Twelve healthy volunteers, 7 women, 30.8±11.6 years of age, collected one stool sample per week over the course of 8 weeks. Date and time of each bowel movement was logged.

For the present study, regular sterile and individually packaged flocked swabs, with plastic applicators (FLOQSwabs™, Copan, Murrieta, CA) were used to collect microbiota samples. The swabs to collect the oral microbiota from the left and right inside of the cheek were collected by one single investigator (C.S.), while the swabs to collect the rectal microbiota were performed by different CTRC nurses on call with a witness present in the room during biospecimen collection. Samples were stored at -80°C until analysis. Also for the present study, saliva was submitted to microbiomics analysis.

For analysis, DNA was extracted from each stool sample, followed by PCR amplification using 16S primers (27f/357R) and 454/Roche pyrosequencing, as described previously [14,15]. Samples were included

in the analysis if a minimum of 1000 sequences were recovered. Bioinformatics analysis was carried out with the QIIME software pipeline [16]. The 16S sequence reads were clustered at 97% similarity using UCLUST [17]. Representative sequences from each cluster were aligned to 16S reference sequences with PyNAST [18], and a phylogenetic tree was inferred with FastTree [19]. Taxonomic assignments were generated using the default method in QIIME, which compares the top three sequence matches in the Greengenes reference database [20].

Plasma and Saliva Metabolomics

Na-heparin plasma and saliva samples (100 µl) from both 48 hour sessions, n=60, were submitted for analysis. The following standards and their concentrations were spiked into each sample: 13C6 Citrate, 7.5ug/mL; 13C4 Fumarate, 2.5ug/mL; 13C5 15N1 Glutamate, 2.5ug/mL; 13C6 Arginine, 1ug/mL; 13C3 Lactate, 2.5ug/mL; 13C6 Glucose, 7.5ug/mL; Lysine d4, 2.5ug/mL. For the sample preparation, 200µl of methanol and 100µl of chloroform were added to 50µL of plasma and samples were then sonicated for 15 minutes. 100µl of chloroform and distilled H_2O were then added and samples were subsequently centrifuged for 7 minutes at 13,000 rpm at 4°C. 200µl was pipetted from the upper aqueous layer of each sample into a new 5as accom tube. Samples were evaporated for 2-24hrs in a speedvac and stored at -80°C until analysis.

LC-MS/MS was performed on a Waters Acquity UPLC coupled to a Waters TQD mass spectrometer (Waters Corporation, Milford, MA, USA). Liquid chromatography conditions and mass spectrometer parameters were based on methods described by Yuan et al.[21], with chromatographic separation of metabolites performed using a Waters BEH Amide (100mm x 2.1mm x 2.5µm) column. Data processing was accomplished using Waters TargetLynx software (version 4.1).

A series of quality control steps was designed to account for individual feature drift in the MS platform, that is features were only retained in the analysis if the coefficient of variation in a pooled sample < 30% across the dataset.

Proteomics

Aliquots of 80 mL plasma samples collected in EDTA during the first 48 hour session, n=30, were submitted to protein analysis using the SomaLogic discovery platform [22]. The SOMAscan™ assay was performed in-house at the Penn Translational Core Laboratories and sent for analysis to SomaLogic, Inc, Boulder, CO. This assay quantifies proteins over eight logs in abundance covering the femtomolar to

micromolar range with 96.5% of all proteins showing a coefficient of variation (CV) < 20%. Briefly, each individual protein concentration is transformed into a corresponding SOMAmer reagent (Slow Off-rate Modified Aptamer) concentration, which is then quantified by standard DNA techniques [23].

Gene Expression

Whole blood (2.5 ml per collection) was collected and stored in PAXgene blood RNA tubes (PreAnalytiX GmbH, Hombrechtikon, Switzerland) at -80°C until use. Total RNA from blood sample tubes collected during session 1 was extracted according to the manufacturer's instruction. 0.4µg RNA was reverse transcribed to cDNA using random hexamer and MultiScribe™ Reverse Transcriptase (Applied Biosystems). The cDNAs were then used as templates for TaqMan gene expression assay using the ViiA™ 7 Real-Time PCR System (Applied Biosystems). Gene expression is reported as relative mRNA level where gene targets of interest were normalized to *GAPDH* and *ACTB* (*β-Actin*). Primers targeted the following genes: *DBP* (D site of albumin promoter (albumin D-box) binding protein, Entrez Gene ID 1628), *ARNTL* (aryl hydrocarbon receptor nuclear translocator-like protein 1, Entrez Gene ID 405), *NR1D1* (nuclear receptor subfamily 1, group D, member 1, Entrez Gene ID 9572), *PER2* (period circadian clock 2, UniGene ID: Hs.58756), and *PTGS2* (prostaglandin-endoperoxide synthase 2 [commonly referred to as cyclooxygenase-2], Entrez Gene ID 5743, Hs.PT.53a.21519633. House-keeping genes were GAPDH (glyceraldehyde-3-phosphate dehydrogenase, Entrez Gene ID 2597, Hs.PT.53a.26759668), and ACTB (beta-actin, Entrez Gene ID 60, Hs.PT.53a.21116011.g). *DBP*, *ARNTL*, *PER2*, and *NR1D1* primers were obtained from Thermo Fisher Scientific, Carlsbad, CA. *PTGS2*, *GAPDH* and *ACTB* primers were obtained from IDT Integrated and Technologies, Coralville, IA.

Smartphone user data

The HIPAA-compliant Ginger.io platform consisted of a mobile phone application and a web dashboard which allowed the researcher to monitor user activity in near real time. The application gathered communication and mobility data through a background process and transmitted data encrypted to firewall protected linux-based servers with access control lists. Study participants used the Android smartphone they owned at the time of study inclusion. Upon invitation users installed the mobile application and logged in with a password provided to them. This initiated the data collection process. The data were stored securely on the mobile device temporarily and sent at regular intervals using the

World Wide Web. In the case that a connection was not available or if a transmission was not successful, the data continued to be stored on the device until the next transmission attempt was successful. In detail, the data collected through the device included call and sms logs including call/sms time, call duration, sms length, and phone number, as well as location information and device usage. Importantly, private information such as actual content of voice calls or sms messages or emails was never accessed, read, recorded, or transmitted. The data were encrypted and transmitted to the server over a secure 128-bit SSL 3.0 connection using the HTTPS protocol. For this purpose, unique keys were generated dynamically for every API request through a unique identifier and client secret that was embedded inside the application for which access was denied by any user or application.

Ginger.io Linux-based servers are protected using a firewall and access control lists (ACLs), and access is restricted to Ginger.io employees and contractors. Superuser activity and researcher activity on the server is logged for security and auditing. The servers are regularly updated and patched with latest security updates to ensure that there are no known threats. These servers host the database where the data is stored. Linux-based servers and access to the servers are restricted to a few users responsible for maintaining and testing the database. For additional security, all the data that has personally identifiable information about the participant (such as e-mail) are stored in a separate database from the one that has user and study data. The passively collected data from the phones as well as the actively-reported survey information is stored in the second database. Phone numbers and other such private information stored in this database are anonymized by hashing over the identifiers so that there is no threat to privacy because the data are not human-readable at this point. During the close out visit of each participant, the Ginger.io application was removed under supervision of study personnel and data recording was discontinued via the dashboard application. The data safety and privacy measures put in place by Ginger.io were reviewed and accepted per Security and Privacy Information Impact Assessment (SPIA) by an Information Security Officer appointed by the Penn Medicine Academic Computing Services. The SPIA formed an obligatory part for the review by the Institutional Review Board.

Sleep survey

A sleep survey was administered daily via the Ginger.io app at 08:00 hours EDT consisting of the questions i) “What time did you fall asleep last night?” and ii) “What time did you wake up?” Answers were given using a time dial function in 12-hour notation with am/pm suffix.

Comparison of Subjective and Objective Sleep Data

We assembled lists of sleep and wakefulness onset times derived from the actigraphy data (using the Cole-Kripke or Sadeh algorithm embedded in ActiLife), and the self-reported sleep survey. Next, we manually aligned the sleep periods from the actigraphy data with the sleep survey. We discarded sleep periods that were present in only one of the data types, and merged fragmented sleep periods derived from the actigraphy data to better reflect the true sleep and wakefulness onset times. Having aligned the sleep periods in this way, we calculated the difference in sleep and wakefulness onset times between the self-reported sleep survey, and the actigraphy-derived results from each of the two algorithms. A one-sample t-test on the differences in sleep/wake times between the two data types was executed to discern differences between self-reported and activity algorithm-base sleep-wake times.

Nutritional Assessments

Remote Food Photography

Intake of food and beverages was collected with the SmartIntake© smartphone app, a validated Remote Food Photography Method® (RFPM) [24,25]. Subjects placed a reference card next to their food/beverage and captured images at 45 degrees of their food/beverage selection and plate waste. If available, subjects scanned the barcode or entered the Price Look-up (PLU) number to identify foods or beverages in the image. Additional food/beverage descriptors were provided by text or voice message which was automatically tagged to the food image. These data and food/beverage images were wirelessly sent by the mobile app to the server-based Food Photography Application©, which was used to manage the data collection process and analyze the food images to estimate energy and nutrient intake. To facilitate data quality and completeness, the SmartIntake© app included Ecological Momentary Assessment (EMA) [26] methodology to remind participants to capture images of the foods and beverages that they consume. These reminders were text messages that are scheduled for delivery at the personalized meal times of the participants. The responses to EMAs were tracked in near real-time, which allowed the research

team to identify quickly when data collection problems occurred. In such cases, (which did not occur in the present study), a back-up method can be used (e.g., a food record or food recall conducted by phone). The SmartIntake© app sends participants food/beverage images and accompanying food identifier data (e.g., barcodes, PLU numbers, food descriptions) to a server located at the Pennington Biomedical Research Center where bionutritionists analyze the images to estimate food/beverage intake based on the Food Photography Application© program. This allows the operator to identify a match for each food from the Food and Nutrient Database for Dietary Studies 5.0 [27] and other sources, such as manufacturer's information and Nutrition Fact Panels, to calculate energy and nutrient intake. Additionally, the operator uses the program to estimate portion size by visually comparing participants' food images to images of foods with a known portion size,i.e., standard portion images. This process relies on existing and validated methodology [24,25,28,29]. The standard portion images are contained in a searchable archive that includes over 8,000 standard portion images. This process results in estimates of food/beverage selected and plate waste, which is used to calculate food intake by difference.

Diet Recall and Food Record

The 24-hour hour diet recall interview was conducted by a bionutrionist using Nutrition Data System for Research (NDSR), version 2014, a computer-based software application developed at the University of Minnesota Nutrition Coordinating Center (NCC) that facilitates the collection of recalls in a standardized fashion [30]. Dietary intake data gathered by interview is governed by a multiple-pass interview approach [31]. Five distinct passes provide multiple opportunities for the participant to recall food intake. The first pass involves obtaining from the participant a listing of all foods and beverages consumed in the previous 24 hours. This listing is reviewed with the participant for completeness and correctness (second pass). The interviewer then collects detailed information about each reported food and beverage, including the amount consumed and method of preparation (third pass). In the optional fourth pass, the interviewer then probes for commonly forgotten foods. Finally, the detailed information is reviewed for completeness and correctness (fifth pass). Dietary supplement use was assessed in conjunction with collection of 24-hour dietary recalls using the Dietary Supplement Assessment Module included in NDSR [32]. Use of all types of dietary supplements and non-prescription antacids were queried in the module.

Participants were asked to record their food intake for three consecutive days covering two weekdays and one weekend day. The 3-day food records were quantified by the bionutritionist using NDSR, version 2014.

The NCC Food and Nutrient Database serves as the source of food composition information in NDSR [33]. This database includes over 18,000 foods including 7,000 brand name products. Ingredient choices and preparation methods provide more than 160,000 food variants. Values for 163 nutrient, nutrient ratios and other food components are generated from the database. The USDA Nutrient Data Laboratory is the primary source of nutrient values and nutrient composition. These values are supplemented by food manufacturers' information and data available in the scientific literature [34]. Standardized, published imputation procedures are applied to minimize missing values [35].

Data Management

Study data were collected and managed using REDCap electronic data capture tools hosted at the University of Pennsylvania. REDCap (Research Electronic Data Capture) [36] is a secure, web-based application designed to support data capture for research studies, providing an intuitive interface for validated data entry, audit trails for tracking data manipulation and export procedures, automated export procedures for seamless data downloads to common statistical packages, and procedures for importing data from external sources.

Data Integration and Visualization

The dplyr (v0.4.3), tidyr (v0.3.1), and lubridate (v1.3.3) packages in R (v3.2.2) were used to manage and integrate study data. For data visualization, the ggplot2 (v2.0.0), scales (v0.3.0), and gridExtra (v2.0.0) packages in R were utilized. Raw data, except blood pressure and heart rate data were square root transformed to allow visualization at scale. To generate the circular plots, the CircOS software package (v0.69) was adapted to represent an entire day as a single circular chromosome, where each position along this chromosome corresponds to a single minute in the 24-hour day (total of 1440 minutes).

Standard Statistical Analyses

Throughout this manuscript, we reported means and standard deviations, if not otherwise noted. We used Wilcoxon paired rank tests to check for significant differences in metabolite and protein abundance between the morning and evening time points. Briefly, we identified each abundance measurement as either morning (0, 24, 48) or evening (12, 36) depending upon its collection time. Next, for each protein/metabolite we calculated the mean morning abundance and evening abundance within each subject. Then, we compared these morning and evening means across all subjects using a Wilcoxon paired rank test (implemented in the *wilcox.test* R function). Within each dataset (ie. plasma proteins, plasma metabolites, and saliva metabolites), we corrected the p-values returned by the Wilcox tests for multiple-testing using the Benjamin-Hochberg method (implemented in the *p.adjust* R function). To assess differences between the sample groups for the microbiome data, we used a multivariate permutational ANOVA (PERMANOVA) test [37] based on pairwise UniFrac distance between samples [38,39]. Subject ID, AM/PM, session 1/2, and left/right side served as independent variables in the test. To control for repeated measures within subjects, we permuted labels for AM/PM, session 1/2, and right/left side only within subjects when computing p-values. Given the resolution of 12 hour intervals for the multi-omics data over the course of 48 hour sessions, we explored and determined that further statistical examination including cross-correlations is not feasible. We found that correlations were unstable with this number of data points especially in the context of multiple testing.

Permutation Test

To address the question of whether patterns of time-of-day fluctuations observed in the metabolome and proteome occur more often than by chance, we employed a permutation analysis. First, we defined two distinct patterns, i.e. M-pattern corresponding to low-high for morning versus evening abundances, and W-pattern displaying high- low abundances for mornings and evenings. Let N be the number of M or W patterns in the data. We then permuted the time points so as to mix up at least one even time point with one odd time point. For each such permutation we counted the number of M and W patterns. Let A be this number averaged over all permutations. If there are meaningful M and W patterns in the data then N should be higher than A. To test this hypothesis we calculated a permutation p-value as the ratio of the number of permutations where there were more M and W patterns than in the unpermuted data to the total number of permutations.

Pathway and Network analysis

To pilot the assessment of transomic linkages, we submitted the plasma and saliva metabolome as well as the plasma proteome data to the Ingenuity Pathway Analysis (IPA, Redwood City, CA). For each combination of metabolite source (plasma or saliva), plasma proteins, session (where available), and time point, we calculated the mean abundance of each metabolite/protein across all subjects and ordered each list of metabolites/proteins by these mean abundances. Next, we took the top half of each list and analyzed the resulting metabolites or proteins using IPA's metabolomics analysis suite or expression analysis suite, respectively. This yielded enrichment *p*-values for the most enriched disease categories among the list of top metabolites/proteins at each time point, in each session, from either plasma or saliva. For those disease categories enriched in only a subset of the data, we entered an enrichment p-value of 1 for all time/session/source combinations where the disease category was absent (ie. showed no enrichment).

For each time point of the proteomic/metabolite data, we then used Fisher's method to combine enrichment p-values between sessions 1 and 2, where available. We next analyzed these temporal patterns in disease category enrichment for evidence of a time-of-day (morning/evening)-dependent effect. Briefly, we used the same permutation methodology to test for enrichment of M and W patterns described above, except in this case we used the Fisher-combined disease category enrichment p-values, rather than molecule abundances. We used STITCH v4.0 (http://stitch.embl.de/) to plot protein/metabolite interaction maps for the enriched metabolites in the disease categories showing significant time-of-day differences.

Circadian Multiresolution Analyses

To identify circadian oscillations as well as signal changes over time, we performed a multiresolution analysis (MRA) of all data derived from the ActiGraph device and the Ginger.io platform. This MRA was based on wavelet transformations and was developed by Price et al. [40]. For the following analyses, we square root transformed the activity and communication data, since they are count data. To begin, we separated the raw data into a set number of component details and a smooth using the mra(w1, wf="mb8", J=5, method="modwt") function in the R waveslim package, where 'w1' is the square root of the raw communication data with missing values replaced using linear interpolation, and the 'J=5'

parameter specifies the maximum level of wavelet details to generate, five levels in this case. We used the "MB(8) from MBDT family of wavelets" [41] as the mother wavelet, where "MB(8)" means using a minimum bandwidth of 8; the "MBDT family" refers to a set of minimum-bandwidth, discrete-time orthonormal wavelets [41].

For data binned into one-hour increments, i.e. the smartphone-derived data, the circadian signal is contained in D_4. Therefore, to retrieve the circadian component of the signal we generated five levels of wavelets, up to D_5. For data binned into one-minute increments, i.e. the actigraph-derived data, the circadian signal is contained in D_{10}. Here, we generated eleven levels of wavelets, up to D_{11}.

After extracting the circadian signal from a dataset, we next produced three metrics: instantaneous amplitude, instantaneous period and instantaneous phase. The first step is to identify the times at which the roots, minima, and maxima occur in the circadian signal. Roots indicate the times at which the circadian signal crosses the x-axis (circadian signal = 0), and the minima and maxima correspond to the troughs and peaks of the circadian signal, respectively. For data with lower sampling resolutions (≥ 30 minutes) we estimated the exact times for these points using linear interpolation. In many cases, this allowed us to calculate instantaneous parameters these interpolated times, rather than the sampled times. For example, if we used interpolation to estimate a peak in circadian signal at 15.3 hours, but we sampled at one-hour resolution, we want values for the instantaneous parameters at 15 and 16 hours. In order to achieve this, we performed a second interpolation step to estimate the instantaneous parameter values at the time points we original sampled. For data with higher sampling resolutions (≤ 4 minutes), the times of these points (roots, minima, maxima) are determined directly from the sampled times, without any interpolation.

Instantaneous amplitude is the absolute difference in circadian signal between a maximum and its preceding minimum (or vice versa). We calculated the amplitude by stepping through each maximum and minimum. If a maximum occurred at time 24:00 and a minimum at time 12:00, we calculated the amplitude as *abs(circadian signal at 24:00 – circadian signal at 12:00)*. If the next minimum occurred at time 36:00, then the next amplitude is *abs(circadian signal at 36:00 – circadian signal at 24:00)*.

Instantaneous period is the length of the period for each complete cycle in the circadian signal. After determining the times for roots, minima, and maxima of the circadian signal, we stepped through each full cycle (i.e. peak-root-trough-root-peak, trough-root-peak-root-trough, or root-peak/trough-root-trough/peak-root), and calculated the difference in time between the end of the cycle and its beginning. If a peak occurred at time 23:00, and the next peak occurred at time 47:00, the instantaneous period for this cycle is 47:00 – 23:00 = 24:00.

Instantaneous phase relates the phase of a signal to a particular time. We assigned the maxima, first root, minima, and second root to the following values in order: 0π, 0.5π, 1π, and 1.5π, respectively. These transformed values define phase values with respect to sinusoidal curves. The time at which the instantaneous phase is equal to 0 corresponds to the peak in circadian signal, since the peak in a cosine occurs at 0 or 2π. Again, we used linear interpolation to determine the phase values for the sample times between the roots, minima, and maxima.

Cosinor Analysis

In addition, we used the cosinor method (reviewed in [42,43]) to fit cosine curves to several of the data types for each subject (i.e. activity, aggregate communication, blood pressure, heart rate, light exposure, and mobility) limited just to the two 48-hour deep phenotyping sessions (Session 1 and 2). These fits yielded MESOR, amplitude, and phase values for each combination of subject, data type, and session.

Variance Correlation Analysis

To examine the relationships between the various datasets we collected, we performed the following analysis.

Preprocess the data

For this analysis we used all of the communication, activity, cardiac measurement, and dietary intake data, and aggregated them at one-hour intervals. We integrated the datasets using hourly aggregates because communication was recorded at a one-hour resolution, while all of the other data were collected at finer resolutions. To produce an hourly summary for activity data and cardiac measurements, we calculated the mean of each measure for a given subject for each one-hour interval. For the dietary data, we summed the total consumption of each nutrient for a given subject for each one-hour interval. We removed the Unreturned Calls measure from the communication set due to the high prevalence of missing values. We also removed rows that included missing measurements. Note,

the cardiac measurements and dietary intake data were only collected during two 48 hour visits, while the other datasets were collected over several months. For this reason, when analyzing the cardiac data we took the subset of the other datasets that include only the time index and subject combinations present in all of the data (i.e. limiting the analysis of all data types just to those 48 hour periods).

After processing, each row in the integrated dataset included the hour-aggregated measures of all variables for a given subject at a given hourly time index, as defined by the hours after the epoch 2014-10-21, 00:00. This dataset included every hour/subject combination present in both the activity and communication datasets. As we intended to perform linear regression, we plotted the distribution of each variable. To improve normality as a condition of linear regression, we transformed the Axis1, Axis2, Axis3, Steps, Luminosity, Activity Vector Magnitude, Mobility, Mobility Radius, Metabolic Rate, Energy Expenditure, and all Circadian Signal variables using a log(x+1) transformation, and applied a square root transformation to the Interaction Diversity, and all of the dietary data.

Statistical analysis of the variance explained between outputs

To evaluate the linear relationships between every pairwise combination of variables in the integrated dataset prepared above, we calculated the linear regression coefficient of determination (R^2), for each pair of variables, using the *lm* function in the R statistical language. We then constructed a heat map of the proportion of variance in each variable (e.g. mobility, luminosity, systolic blood pressure) explained by every other variable. We performed calculations and produced heatmaps for data from all four months of collection for communication, activity and environment variables. We also used the subsets that contained the biometric visits to produce a heatmap that included these variables along with the blood pressure, heart rate, and dietary intake variables. In addition to the R^2 values, we also examined the p-values produced by the linear regression analysis. We produced heatmaps for the p-values as well, but only displayed data for those p-values that remained significant following a Bonferroni correction.

Principal Components Analysis

To visualize variance effects in the multi-omics data, we used principal components analysis (PCA). Briefly, we prepared data matrices for each of the multi-omic datasets for all subjects and clinic visits by imputing (for any missing data points) and normalizing into unit variance before PCA fitting. The initial

PCA fitting and plotting guided outlier removal if necessary (for example HCR009-12hr-Visit 1 for proteomics), which we followed by refitting. We then used PCA scores as latent variables for correlation analysis with the other data types. For dietary intake, we broke data from each clinic visit into chunks, at the midpoint between sampling times, yielding a total of ten chunks per visit. We then summed the macronutrient intake within each chunk. For all PCA, we used Python's scikit-learn module (version 0.17.1).

Contribution of Time and Subject to Variability in Measurements

Standard methods of classical statistics were used to assess the contribution of time and subject (separately) to variability in the various datasets. For these analyses the same datasets were used as the variance correlation analysis, and the same pre-processing steps were performed. Additionally, the proteomics, metabolomics, and microbiome datasets were used in these analyses. The model is formalized as follows. First, observations were grouped by clock hour (0-23) or time of day (morning, evening) when assessing time; or by subject when assessing inter-subject differences. The contribution of the grouping variable (time or subject) to variance was computed as the following sum of squared differences:

$$SS_{group} = \sum_{i=1}^{n} (\overline{Y}_i - \overline{Y})^2$$

where i is a value of the grouping variable (i = hour for time or subject number for subject), n is the number of values for the grouping variable (e.g. n=6 for subject; n=24 for clock hour), $\overline{Y_i}$ is the mean measurement for the ith value of the grouping variable (e.g. average of all blood pressure measurements from hour three), and $\overline{Y}$ is the mean measurement value across all of the data. Note, the SS_{group} is equivalent to the lack of fit sum of squares of the data to a horizontal regression line [44]. The greater the effect time or subject has on the variability in the data, the greater SS_{group} will be. Next, the total variability was calculated as the following sum of squared differences:

$$SS_{total} = \sum_{j=1}^{m} (Y_j - \overline{Y})^2$$

where *j* here refers to a specific measurement (e.g. bp measurement from subject one, at hour three, on day two), *m* is the total number of measurements, Y_j is the value of the jth measurement, and $\overline{Y}$ is again the mean across all of the data. Lastly, the grouping variable's contribution to the total variance is captured in the following ratio:

$$Contribution\ to\ Variance = \frac{SS_{group}}{SS_{total}}$$

This is analogous to the R^2 calculation performed in linear regression modeling, where the values of the grouping variable serve as discrete values of the predictor variable. In this case, we used the mean values across the ith value of the grouping variable as the fitted value of the regression curve, in order to account for a non-linear relation between variables. This was possible because the predictor variables are discrete and there are replicate observations of the outcome variables at each value of the predictor variables. The above calculation was then repeated for each type of data: blood pressure measurements, abundance levels of individual proteins/metabolites, actimetry, etc.

Open Source Data Sharing

De-identified raw and aggregate data will be shared via Zenodo, https://zenodo.org/, a free option for hosting large datasets ("Big Data") in a citeable form. Zenodo is hosted by CERN (Conseil Européen pour la Recherche Nucléaire), the European Organization for Nuclear Research in Geneva, Switzerland, which is backed by significant financial resources, retains data for the lifetime of the repository (currently secured until at least 2037), defines that all uploaded content remains the property of the parties prior to submission, allows all data formats, and keeps data files in multiple replicas in a distributed file system.

Supplemental Results

Human Subjects and Safety

A cohort of eight healthy volunteers was enrolled in this study. Six volunteers completed the study, all males, 32.3±3.6 years of age, BMI 25.2±3.4 kg/m2, Table S 1), while two dropped out (inclusion criteria were not met). Subjects screened negative for vitamin intake except as follows: one subject, HCR003, recorded oral intake of an over-the-counter multivitamin formulation during an episode of "scratchy throat" (Airborne Effervescent Tablets containing among others 1000 mg vitamin C; dose of 1 tablet/day over 3 days) which did not coincide with either of the two 48-hour sessions of deep phenotyping. Subjects screened negative in urine tests for drugs and cotinine (a nicotine metabolite) as well as by a platelet aggregation assay administered to assess compliance during screening at the beginning of each 48 hour session. The safety laboratory assessments of blood count and blood chemistry at screening and the exit visit were normal in the chart review. Serious adverse events did not occur. A total of nine adverse events were recorded; all were mild in severity and resolved. Per the Munich Chronotype Questionnaire (MCTQ), 3/6 subjects were rated as normal chronotype, 2/6 as slight late, and 1/6 as slight early. 5/6 subjects pursue regular work starting and ending the work day on average at 08:36±00:33 and ending 17:54±02:33, thus spending 9.3±2.8 hours at work. One subject, HCR003, indicated both regular work hours (09:00 to 16:00) and shiftwork in the MCTQ, however, during study enrollment shiftwork was not reported and rhythms of activity counts, ambient light intensity and self-reported sleep times reflected a daytime work schedule. 4/6 subjects spend 15 minutes each way walking or biking to work, while one subject commutes for 30 minutes each way in a vehicle. One subject did not provide information. In the chart review, laboratory tests assessing kidney function were within the normal range for both sessions 1 and 2. (Table S 2, and Table S 3).

Data Volume, Handling and Integrity

Per clinical study protocol, the collection of approximately 7.5 billion samples was anticipated via remote sensing, while around 6,000 clinical samples were planned to be collected. Processing of remote sensing data, that is, re-integration of activity and ambient light intensity data to larger 60-second epochs as described below, reduced this data set to about 2.1 million data points, while analysis of clinical samples increased this data set to about 106,000 data points. Thus, ~2.2 million data points were used for both visualization and further bioinformatics analysis including efforts to integrate these multidimensional datasets.

Ambulatory Blood Pressure Monitoring

Blood pressure shows a strong diurnal rhythm in this cohort of healthy participants. The mean systolic and diastolic blood pressure during waking hours was 126.1±10.6 mmHg and 78.5±9.1 mmHg, respectively, which dropped at night to 106.8±11.6 mmHg and 58.8±10.2 mmHg, respectively. This nocturnal dip lowered systolic and diastolic blood pressure by 20.7±3.9 and 20.7±3.3 mmHg, respectively. A mean ratio SBP_{asleep}/SBP_{awake} of 0.85 classifies this as a dipping phenotype as the ratio falls into the category of >0.8 but ≤0.9 [45,46]. The largest nocturnal dip of 24.2±2.7 bpm was observed for HCR009 (from 80.1±11.8 to 59.1±14.6 bpm), while the smallest nocturnal dip of 13.1±7.7 bpm was evident for HCR003 (66.8±12.1 to 54.4±7.9 bpm). Mean arterial pressure (MAP) dropped by 19.4±3.2 mmHg, while pulse pressure (PP) remained stable during wake and sleep hours. Diurnal patterning is also discernible for heart rate readings as shown in Figure 2 and Table S 4 and Table S 5. Heart rate reached on average 74.0±13.0 bpm during wake times in session 1 and 2, dropping by 16.5±6.6 bpm to 58.8±11.9 bpm during self-reported sleep times. (Table S 4, Table S 5).

Activity

Wrist actigraphy collected high resolution data for the duration of four months. Wear time was high (96%, 94.8%, 95%, 96.6%, 97.5%, and 86.5% for HCR001, HCR003, HCR004, HCR006, HCR008 and HCR009, respectively. The vector magnitude data we report here is well within the range of physical activity intensities found in people over a broad age range [47]. On average, activity of the six participants amounted to 1904.0 (range: 0-55757.7) counts • min^{-1} during wake time, falling to 307.2 (range: 0-29642.7) counts • min^{-1} during self-reported sleep times (Table S 6). Note that maximum nocturnal activity counts are confounded by wake time activity due to a small number of erroneous self-reported sleep times (detailed below). Highest daytime activity counts of 2233.9 (range: 0-37935.8) counts • min^{-1} were achieved by HCR001, while 1599.2 (range: 0-16080.0) counts • min^{-1} for HCR006 was the lowest observed. This was inverse for the self-reported sleep times; 176.9 (range: 0- 26259.6) counts • min^{-1} for HCR001 was the lowest, while 427.4 (range: 0-12667.9) counts • min^{-1} for HCR006 was the highest observed in this cohort. This pattern showed consistency in this cohort. Ranking highest to lowest mean activity during daytime ordered the subjects as HCR001, HCR008, HCR003, HCR009, HCR004, and HCR006; ranking highest to lowest mean activity during self-reported sleep reversed this order to HCR006, HCR004, HCR009, HCR008, HCR003, and HCR001. Activity counts during sleep might indicate periods of wakefulness [48].

The comparison of the six male participants to the NHANES cohort suggest that our study subjects i) differed in their activity profile (more percent counts for sedentary, moderate, vigorous and very vigorous, less for light, and equal percent counts for the category lifestyle); ii) displayed higher energy expenditure; iii) within narrow defined age brackets; iv) while following the distribution of BMI.

Ambient Light Intensity

Ambient light intensities over the course of four months averaged in this cohort to 42.3 (range:0-6500) lux • min^{-1} during wake hours and 2.7 (range: 0-5853; maximum confounded by daytime light due to a small number of erroneous self-reported sleep times as detailed below) lux • min^{-1} during sleep hours. The daytime level of illumination corresponds to intensities reported for indoor environments during overcast days [49], which are expected in Philadelphia for the winter months of October through March. Nighttime illumination reached levels corresponding to moonshine [50]. A note to consider is that intensity readings might be confounded during winter months by long sleeves covering the light sensor on the wrist-worn device. A substantial higher mean light intensity were evident for HCR001 during wake hours, 119.3 (range 0-6500) lux • min^{-1}, compared to the remainder of the cohort. These high values coincided with the times when HCR001 was exposed to sunny environments when traveling to Latin America.

Microbiome

In a separate pilot study, 12 healthy volunteers collected weekly stool samples over a period of eight weeks. The relative abundances of the phylum Bacteroidetes suggests two peaks during the course of a 24-hour day, one in the morning (circa 06:00-08:00), and one in the evening (circa 20:00-22:00) despite the observed variability. The relative abundances of the phylum Firmicutes ran inversely to Bacteroidetes, as these phyla are the two most abundant bacterial communities in the human gut. These data framed the hypothesis that the human microbiome varies with time of day (Figure S 6).

In the present study, time-of-day dependent differences in the relative abundances of several genera were most pronounced in the buccal mucosa, less so in saliva and rectal mucosa. The abundance of *Streptococcus* increased in buccal mucosa from 37.5±9.8% abundance in the morning to 63.9±13.0 % in the evening compared to 23.2±6.5% and 27.6±11.3%, when sampled in saliva. *Veillonella* showed an inverse pattern, decreasing from 7.9±3.1% to 2.6±1.4% in buccal mucosa and 13.2±5.1% to 8.7±4.1% in

saliva. In the rectal mucosa, *Peptoniphilus* decreased from 4.6±4.2% to 2.1±2.0%; while *Ruminococcaceae* grew from 5.2±5.1% to 6.9±5.2.

To assess community-level differences in the microbiota, we carried out a PERMANOVA analysis [37] of unweighted UniFrac distances [38] for the buccal mucosa samples, using subject ID, morning/evening sampling, first/second 48-hour session and left/right cheek side as variables. Subject ID accounted for the highest degree of variability, R^2 of 0.183, p=0.001; which was followed by morning/evening, R^2 of 0.035, p=0.007. Session (R^2 of 0.025, p=0.064) and cheek body side (R^2 of 0.014, p=0.837) added a low degree of variability. This pattern was consistent for the weighted UniFrac distance analysis [39]. Subject ID accounted for the highest degree of variability, R^2 of 0.329, p=0.001, followed by morning/evening, R^2 of 0.29, p=0.001. Session (R^2 of 0.025, p=0.06) and cheek body side (R^2 of 0.009, p=0.3) added a low degree of variability.

The rectal flora revealed a similar pattern in the PERMANOVA analysis. In the unweighted UniFrac distance analysis, most of the variability was attributable to subject ID, R^2 of 0.246, p=0.001; followed by morning/evening, R^2 of 0.018, p=0.002, and then by session, R^2 of 0.018, p=0.005. This was consistent for the weighted analysis: subject ID (R^2 of 0.417, p=0.001), followed by morning/evening (R^2 of 0.031, p=0.002), and session (R^2 of 0.025, p=0.015).

Between-session and time-of-day differences were also discernable in the UniFrac distances of the salivary microbiome. In the PERMANOVA test of unweighted UniFrac distances, using subject ID, morning/evening sampling, and first/second 48-hour session as variables, subject ID accounted for the highest degree of variability, R^2 of 0.324, p=0.001; which was followed by session, R^2 of 0.019, p=0.004; and morning/evening, R^2 of 0.017, p=0.009. Interestingly, the between-session effect was not observed for the weighted UniFrac distance analysis, R^2 of 0.010, p=0.281. Again, subject ID accounted for the highest degree of variability, R^2 of 0.521, p=0.001; while the variability attributable to morning/evening, R^2 of 0.039, p=0.002 was higher compared to the unweighted analysis. Please see Figure S 7.

Metabolomics

In these screens a total of 166 and 250 features were identified in plasma and saliva samples, respectively. This includes features for which corresponding metabolites could not be identified; a respective total of 41 (24.7%) and 59 (23.6%) unknown features. These features, identified by their mass transitions, were included in data visualization and analysis.

Cortisol showed the expected diurnal variance, being higher in plasma at 1.3±0.4 in the morning than 0.8±0.2 [normalized intensities] in the evening for this cohort of n=6. The averaged time series data (for both sessions) demonstrates this: values were 1.4±0.5 in the morning of day 1 (0h), 0.8±0.3 in the evening of day 1 (12h), 1.2±0.2 in the morning of day 2 (24h), 0.8±0.2 in the evening of day 2 (36h), followed by 1.2±0.3 in the morning of day 3 (48h). In comparison, cortisol levels in saliva show much higher variability. The difference in means, however, is consistent with a diurnal pattern for the averaged morning versus evening values, a respective 0.9±0.9 versus 0.6±1.3. The temporal differences in the metabolites suggested by visual inspection failed to attain significance in the basic Wilcoxon paired rank test including Benjamini-Hochberg correction. However, the number of time-of-day fluctuations following M or W patterns was significantly higher in the unpermuted salivatory metabolome dataset (n=121 for the first 48h-session, p=0.009; n=82 for the second 48h-session, p=0.009) compared to the average number of M and W patterns over all permutations (n=53 for the first 48h-session; n=49.2 for the second 48h-session). This was also observed for the plasma metabolome during the second 48h-session (n=94 M and W patterns in unpermuted data, n=60 average number, p=0.009) albeit not for the first 48h-session (n=89 M and W patterns in unpermuted data, n=58 average number, p=0.1215).

Visual inspection suggests that several other features showed time-of-day dependent differences in plasma and saliva with the following patterns: i) strong time-of-day dependent differences are in-phase for all subjects and sessions, for example, for biphosphoglycerates (intermediate in glycolysis) in saliva; ii) time-of-day dependent differences are in-phase for some subjects across both sessions, for example, myo-inositol (glucose isomer with insulin-sensitizing effects) in plasma for HCR001, HCR006 and HCR009, while other subjects show dampened or opposing patterns (HCR003, HCR004, and HCR008); iii) time-of-day dependent differences are in antiphase for session 1 versus session 2, for example, imidazoleacetic acid (histamine oxidation product) in plasma of subject HCR008; and iv) global patterns between

subjects, for example, dampened time-of-day dependent differences for the salivary metabolites for HCR009 compared to HCR008.

Proteomics

Quantitative multiplex proteomic SomaLogic analysis identified 1141 proteins enriched for cardiovascular diseases, diabetes mellitus and other disease entities. Time-of-day dependent differences in protein concentrations, quantified as relative fluorescent units (RFU), were observed with a high degree of variability between morning and evening, for example, VEGFC (Vascular Endothelial Growth Factor C) showed 552.7±85.7 in the morning versus 678.1±262.2 in the evening; HAMP (Hepcidin) 3974.1±1636.0 versus 6614.7±2109.8; MB (myoglobin) 934.9±204.0 versus 706.7±141.8; or HSP90AA1/HSP90AB1 (heat shock protein HSP 90-alpha) 15290.5±5032.9 versus 23619.6±21052.8. Temporal differences in the proteins as suggested by visual inspection failed to attain significance in the Wilcoxon paired rank test including a Benjamini-Hochberg correction. Furthermore, the number of time-of-day fluctuations following M or W patterns was not significantly higher in the unpermuted plasma proteome dataset (n=450 for the first 48h-session, p=0.5607) compared to the average number of M and W patterns over all permutations (n= 567 for the first 48h-session).

Pilot Network Mapping of Metabolites and Proteins

To search for temporal patterns at the regulatory network level, we used Ingenuity Pathway Analysis (IPA) to determine which disease categories and pathways showed enrichment in our protein and metabolite data at each time point. We saw the number of disease categories with time-of-day fluctuations in IPA enrichment following M or W patterns was significantly higher in unpermuted plasma and saliva metabolome datasets (n=340 for plasma, p=0.028; n=344 for saliva, p=0.009), compared to the average number of M and W patterns over all permutations (n=116.6 for plasma; n=121.9 for saliva). For the plasma and saliva metabolome datasets, we identified the disease categories showing the greatest differences in enrichment between the morning (0h, 24h, 48h) and evening (12h, 36h) time points. These disease categories were abdominal neoplasm, malignant solid tumor, and chronic inflammatory disorders. For the protein dataset, we also observed a higher number of pathways with IPA enrichment following M and W patterns in unpermuted data (n=251, p=0.009), compared to the average across all permutations (n=120.9). The proteins tended to enrich more for functional

categories, rather than disease annotations. Notably, several categories related to cancer and inflammation, e.g. metastatic colorectal cancer and inflammatory bowel disease, were identified. Please see Figure S 5.

Gene Expression

The relative expression of selected molecular clock genes showed some differences between morning and evening in whole blood cells. *ARNTL (BMAL1)*, when normalized to GAPDH, is on average expressed more highly in the evening, 23.3±13.5, than in the morning, 17.9±9.4, n=6. This pattern is evident also in the averaged time series data; 19.1±10.2 in the morning of day 1 (0h) versus 23.4±13.4 in the evening of day 1 (12h), followed by 18.0±10.4 in the morning of day 2 (24h) and 23.2±14.2 in the evening of day 2 (36h), and 16.7±8.3 in the morning of day 3 (48h). In the subject-level time series data HCR001, HCR004, HCR006 and HCR009 show this most convincingly for session 2 but not for session 1. HCR003 and HCR008 show high variability. Interestingly, these patterns are less evident for *ARNTL* when normalized to *ACTB (ACTIN)*, where relative expressions in the morning, 5.2±2.0, correspond to the evening, 5.8±2.5.

Notably, average expression of the reference (housekeeping) genes *GAPDH* and *ACTB* did not differ significantly between morning, 20.5±0.7 and 18.8±0.8, respectively, and evening 20.6±0.7 and 18.7±0.8, respectively. However, individual time series data show variable time-of-day and between-session differences for both actin and *GAPDH*. Stable temporal expression of actin is notable for HCR004, HCR006, and HCR008.

NR1D1, normalized to *GAPDH*, was lower in the morning, 37.7±20.3, compared to evening, 45.6±24.1; however, a much smaller difference was evident when the data were normalized to *ACTB*, 10.9±3.4 versus 11.5±3.8. This pattern was also evident for *DBP*. When *DBP* expression was normalized to *GAPDH*, the morning, 86.9±54.0, was lower compared to the evening, 95.3±55.7, but not different when normalized to *ACTB*, 24.6±8.5 in the morning versus 23.8±8.2 in the evening.

Time-of-day dependent differences in *PER2* expression, normalized to *ACTB*, can be discerned for HCR003 and HCR004, but are less clear for the remaining subjects, so that the group means are not significantly different between morning and evening.

Social Sensing

The mobile app Ginger.io collected social sensing data from smartphones operating on Android OS. The continuous data stream was at times disrupted due to software conflicts between Ginger.io and other smartphone applications. These disruptions commonly resulted from phone updates that interfered with the Ginger.io app settings. These events were usually noted and resolved in a timely fashion by the researcher's access to the Ginger.io dashboard. However, this caused stretches of missing data ranging from several hours to several days, most prominent for HCR008 in late December 2014. For subject HCR001, data were not received during travel outside the US in late November and December 2014 (staying within Eastern Standard Time ±1 hour). HCR009 intentionally did not provide user data in late December 2014 and early January 2015. The pattern of missing data is visualized in Figure S 1.

This cohort of six participants placed and received a total of 1774 calls and sent and received 10,794 text messages over the course of four months, averaging to 295.7±268.4 calls and 1799.0±1712.3 text messages, each. A total of 64,924 location signals were collected from the smartphone's GPS unit, an average of 10,820.7±3368.0 signals per participant.

In the four months of observation, study participants on average used text messages (16.0, range: 0-166 text messages) more frequently than phone calls (2.7, range: 0-48 calls) to communicate during wake times. Communication during self-reported sleep times was minimal (0.1, range: 0-10 calls and 0.8, range: 0-75 text messages). Aggregate communication was 18.7 (range: 0-166) calls and text messages during the wake hours of one day and 0.9 (range: 0-84) during self-reported sleep hours of one night. We found three types of aggregate communication among the six subjects. Four participants preferred text messages over phone calls (HCR001, HCR004, HCR006, and HCR009); one participant preferred phone calls (HCR003); and one (HCR008) used both phone calls and text messages with similar frequency. This level of communication was on average achieved by interacting with 7.2 (range: 0-71) persons during the wake hours of one day. This interaction diversity ranged from 1.7 (range: 0-8) persons for HCR003 to 23.7 (range: 1-71) persons for HCR009 during the wake hours of one day.

Participants were recruited from local communities; hence mobility was low with 1.6 (range: 0-30.6) miles during the wake hours of one day (0.2, range: 0-6.7 miles recorded during self-reported sleep times). The mobility radius amounted to 6.8 (range: 0-193.4 miles during the wake hours of one day

(and 0.2, range: 0-19.3 miles during self-reported sleep). The highest daytime mobility of 2.2 (range: 0-30.6) miles was achieved by HCR001; lowest of 1.1 (range: 0-5.8) miles by HCR003. Daytime mobility radius was highest for HCR003 (11.3, range: 0-193.4 miles), and lowest for HCR004 (4.6, range: 0-79.2 miles).

The average call duration during the four months of remote sensing was 820.4 (range: 0-16590 seconds (or 13.7, range: 0-276.5 minutes) during the wake hours of one day; and the average length of a text message amounted to 868.0 (range: 0-14840 characters. The number of unreturned calls was on average 0.4 (range: 0-8); and the number of calls unanswered, i.e. “missed interaction”, was 0.6 (range: 0-9). (Table S 6)

Sleep Survey

Participants were administered an average of 117.8±3.5 surveys during the study enrollment of four months. These were answered in 100% of cases by subjects HCR003, HCR004 and HCR006. High response rates were also achieved by HCR001 (95.7%), HCR009 (81.0%), and HCR008 (71.9%). The self-reported in-bed times started on average at 00:54±2.5 hours and ended at 08:33±2.1 hours, resulting in sleep duration of 7.6±2.9 h per night. Onset of sleep ranged from 22:50±2.7 hours (HCR008) to 02:11±2.7 hours (HCR006). The average sleep phase lasted until 07:11±2.2 hours (HCR008) as the earliest to 10:22±1.8 hours (HCR006) as the latest observed in this cohort.

Screening the self-reported sleep times for potential erroneous time intervals, misdialing ‘am’ and ‘pm’ for the US American 12-hour clock on the smartphone survey, resulted in 25 calls for abnormal sleep periods. This corresponds to 3.9% of surveys answered by the cohort of n=6 over the 4 months of administration, spanning from 0.8% (1 abnormal sleep period) for HCR3 to 7.3% (6 abnormal sleep periods) in HCR008. These abnormal sleep periods have to be considered as contributors to the incidence of communication, albeit low, during self-reported sleep (see above).

Subjective versus Objective Sleep-Wake Times

Overall, subjective and objective In-Bed-Times (sleep times) and Out-of-bed times (wake times) were in agreement for the Cole-Kripke and Sadeh data, respectively, in our cohort of six volunteers. However, the In-Bed-Times from the Sadeh data were estimated about 30 minutes later than the self-reported sleep times (-27.3, 95% confidence interval -40.4 to -14.2, p=4.7E-05, n=616 observations), and the Out-of-bed times from the Cole-Kripke data about 20 minutes later (-21.2, 95% confidence interval -29.6 to -12.8, p=8.9E-07, n=628 observations). Please see Figure S 8.

On the subject-level, subjective and objective data are mostly in agreement for HCR001 and HCR008. In detail, in-bed times were estimated about half an hour earlier from the Cole-Kripke data than from the survey data (28.4 min, 95% confidence interval 12.1 to 44.4, p<0.0007, n=108) for HCR001. Out-of bed times were estimated about half an hour earlier from the Sadeh data than from the survey data (33.9 min, 95% confidence interval: 5.9 to 61.8, p=0.018, n=80) for HCR008. For HCR003 and HCR006, actigraphy data defined sleep or wake times were later compared to the self-reported data ranging from about 20 to 50 minutes and 60 to 150 minutes, respectively, reaching statistically significant differences. In HCR004, the out-of bed times from the Cole-Kripke data were estimated to be about 40 minutes later than survey data (-39.7, 95% confidence interval -55.6 to -23.7, p=2.84E-06, n=111), while the Sadeh data estimated the out-of bed times about 40 minutes earlier (42.5, 95% confidence interval 19.3 to 65.6, p=0.0004, n=105).

Nutritional Assessments

The dietary food intake was assessed per 3-day food records and 24-hour recalls as well as logged via remote food photography during each of the 48 hour sessions. Only two out of the six subjects were sufficiently compliant to provide the 3-day food record, five subjects were available for the 24-hour dietary recall interview, and all six subjects used the SmartIntake mobile app regularly to document meals and beverages.

Based on the remote food photography method (RFPM), energy intake shows highly variable diurnal patterns among the participants of this cohort. While HCR001 adheres to regular times for breakfast, lunch and dinner with no additional food intake in between, other participants, foremost HCR008, do show clustering around typical food intake times, but also report frequent energy intake during times

between major meals. Consequently, variable patterns of self-reported intake of consumed food weight, protein, total fat, carbohydrates, caffeine/theobromine, total sugars, total dietary fiber, saturated fat, monounsaturated fat, and polyunsaturated fat are evident over the course of 24 hours, corroborating the fluctuating eating patterns in Western society [51].

RFPM including its ecological momentary assessment (EMA) approach achieves estimates on energy intake comparable to the gold standard of doubly labeled water [24], a particular advantage over other self-reported dietary assessment tools known to underreport energy intake [52]. In our study, we see this for the cases where data from the 24-hour recall and RFPM were collected for identical days. Energy intake estimated from the 24-hour recall was 29.6±10.4% lower than from RFPM. In detail, HCR004 consumed 1795.2 kcal (24-hour recall) versus 2567.5 kcal (RFPM) on his first day of the 1st 48h-Session; HCR009 consumed 1492.8 kcal (24-hour recall) 2478.0 kcal (RFPM) on his first day of the 1st 48h-Session; and HCR009 consumed 1978.5 kcal (24-hour recall) versus 2440.0 kcal (RFPM) on his first day of the 2nd 48h-Session.

Acrophase Alignment

We conducted a phase analysis using the cosinor method across the different output variables. We used the recent publication by Roveda E et al. [53] to infer chronotypes for our participants from the actigraphy data using the proposed cutpoints compared to the MCTQ-derived chronotypes (Table S 7).

The graph in Figure S 9 visualizes how the acrophase computed from actigraphy data compares to the acrophases computed for communication, light exposure, mobility, blood pressure and heart rate. Notably, acrophases from these outputs are closely in-phase (second session of HCR008 and HCR009 in particular) or are more dispersed (first session of HCR003 in particular).

Legends Supplemental Figures

Figure S 1. Data Integrity

The top part (A) illustrates the integrity of each individual participant's data set collected over four months (October 2014 to March 2015) shown color-coded for activity, aggregate communication, mobility and sleep survey. The bottom (B) displays the integrity of data collected over the two 48-hour sessions which is color-coded for blood pressure/heart rate, metabolomics, proteomics, and microbiomics. White areas indicate missing data. Note that proteomics were intentionally run for the first 48-hour session only.

Figure S 2. Long-term Remote Sensing – Subject HCR001

(a) Vertical panels display the following data for subject HCR001: activity [square root of vector magnitude], aggregate communication [square root of the sum of counts of phone calls and text messages], interaction [square root of counts•min^{-1}], light intensity [square root of lux•min^{-1}], mobility [square root of miles] and mobility radius [square root of miles] sampled over approximately four months. Each row depicts one week, starting on a Monday. Self-reported sleep times are marked as grey bars.

(b) During the 4-month interval of remote sensing subject HCR001 travelled to destinations in Latin America. This explains the higher mean light exposure compared to the winter months in Philadelphia, PA.

Figure S 3. Nutritional Assessments

Time-of-day dependent intake of a) consumed food weight, b) protein, c) total fat, d) carbohydrates, e) caffeine/theobromine, f) total sugars, g) total dietary fiber, h) saturated fat, i) monounsaturated fat, and j) polyunsaturated fat for all subjects during session 1 (outer circle) and session 2 (inner circle). The data in each session track display nutrient intake for two full days of each session. 24-hour clock times are listed around the edge of the plots, with "00" corresponding to midnight, and "12" corresponding to noon. Dots are color-coded by subject and indicate the level of nutrient intake at the corresponding clock time. Sleep spans are also color-coded by subject and are indicated using the bars below each of the corresponding session.

Figure S 4. Multiomics Principle Component Analysis

The principle component analysis plots show the metabolomic-proteomic-microbiomic signatures color-coded for each participant. These plots were generated from data collected during the first 48hrs session.

Figure S 5. Networks

These plots visualize the networks as defined by the Ingenuity Pathway Analysis. S5a: abdominal neoplasm, plasma; S5b: chronic inflammatory disorder, plasma; S5c: abdominal neoplasm, saliva; S5d: malignant solid tumor, saliva. Red lines indicate metabolite-metabolite interactions, blue lines indicated protein-protein interactions, and green lines indicated metabolite-protein interactions. The thickness of the lines is determined by the amount of evidence for the interaction present in the STITCH databases (http://stitch.embl.de/); thicker lines indicate greater confidence.

Figure S 6. Time-of-Day Differences in Microbial Abundances (separate pilot study)

The relative abundance of the phyla bacteroidetes (top) and firmicutes (bottom), the two most abundant bacterial communities in the human gut, are displayed over a 24 hour time course (abscissa displayed using military hours). Each dot represents the time and abundance of a single stool sample with the central tendency indicated by a polynomial fit in red.

Figure S 7. Microbiomics UniFrac Distances

Time-of-day dependent differences (morning in red; evening in blue) are shown for unweighted (left) and weighted (right) UniFrac distances as principal coordinate analysis (PCoA) for swab samples collected from the buccal (top) and rectal (bottom) mucosa as well as for saliva (center) for session 1 (circles) and session 2 (triangles). Note the missing data for buccal and rectal microbiota for two subjects (HCR003 and HCR006).

Figure S 8. Comparison of Subjective and Objective Sleep-Wake Times

Time difference between sleep survey and Actigraph-derived sleep-wake data displayed for both sleep scoring algorithms Cole-Kripke (top) and Sadeh (bottom) graphed versus number of sleep periods. A positive time difference on the abscissa indicates that the actigraphy data defined sleep or wake times are earlier than the times collected from the sleep survey (see insert on the right).

Figure S 9. Acrophase Alignment

Comparison of acrophases for behavioral, environmental and cardiovascular outputs [activity, aggregate communication, diastolic blood pressure, heart rate, mean arterial pressure, systolic blood pressure,

light exposure, mobility and mobility radius] within each subject and 48-hour session 1 and 2. Acrophases in clock time (h, abscissa) were calculated per session from the two daily cycles covered by 48-hour observation time.

Supplemental Figures

Figure S 1 – Data Integrity

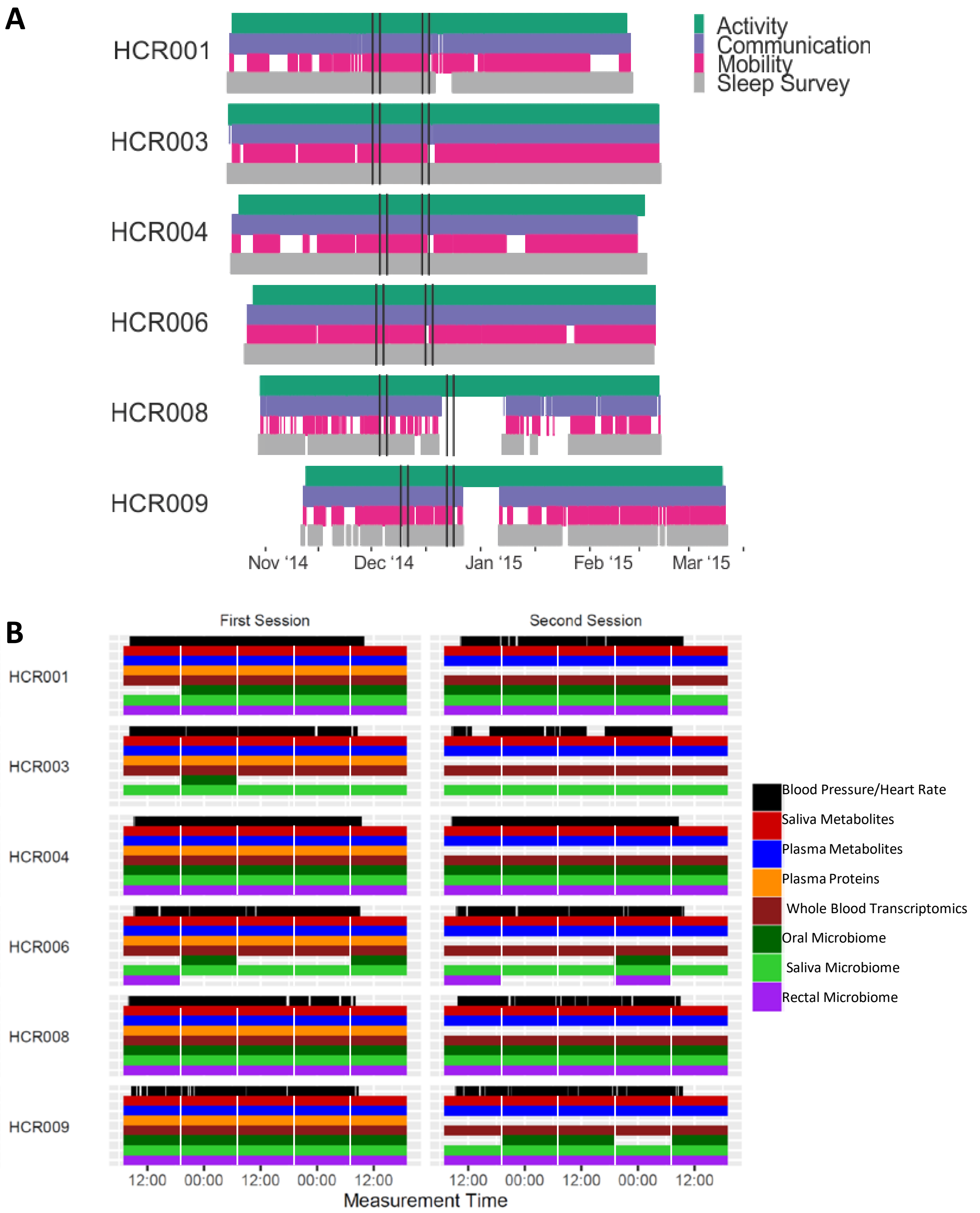

Figure S 2a – Long-term Remote Sensing – Subject HCR001

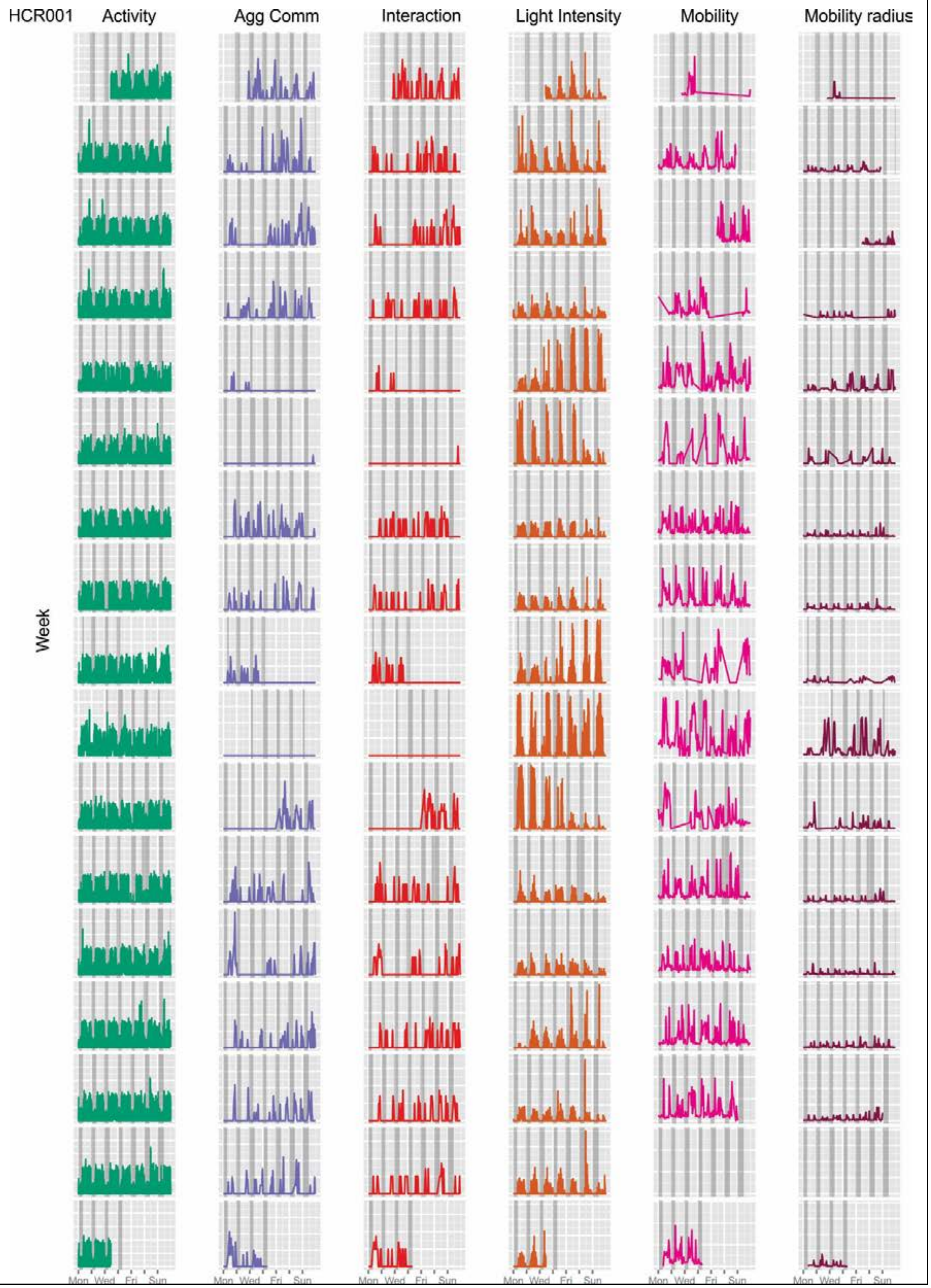

Figure S 2b – Light Exposure HCR001

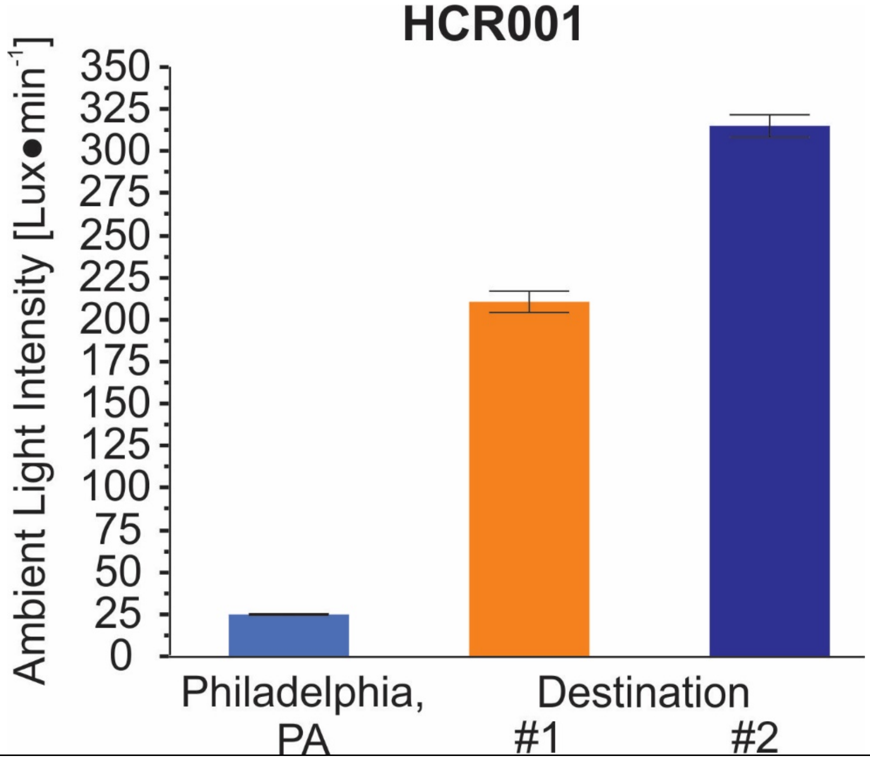

Figure S 3 – Nutritional Assessments

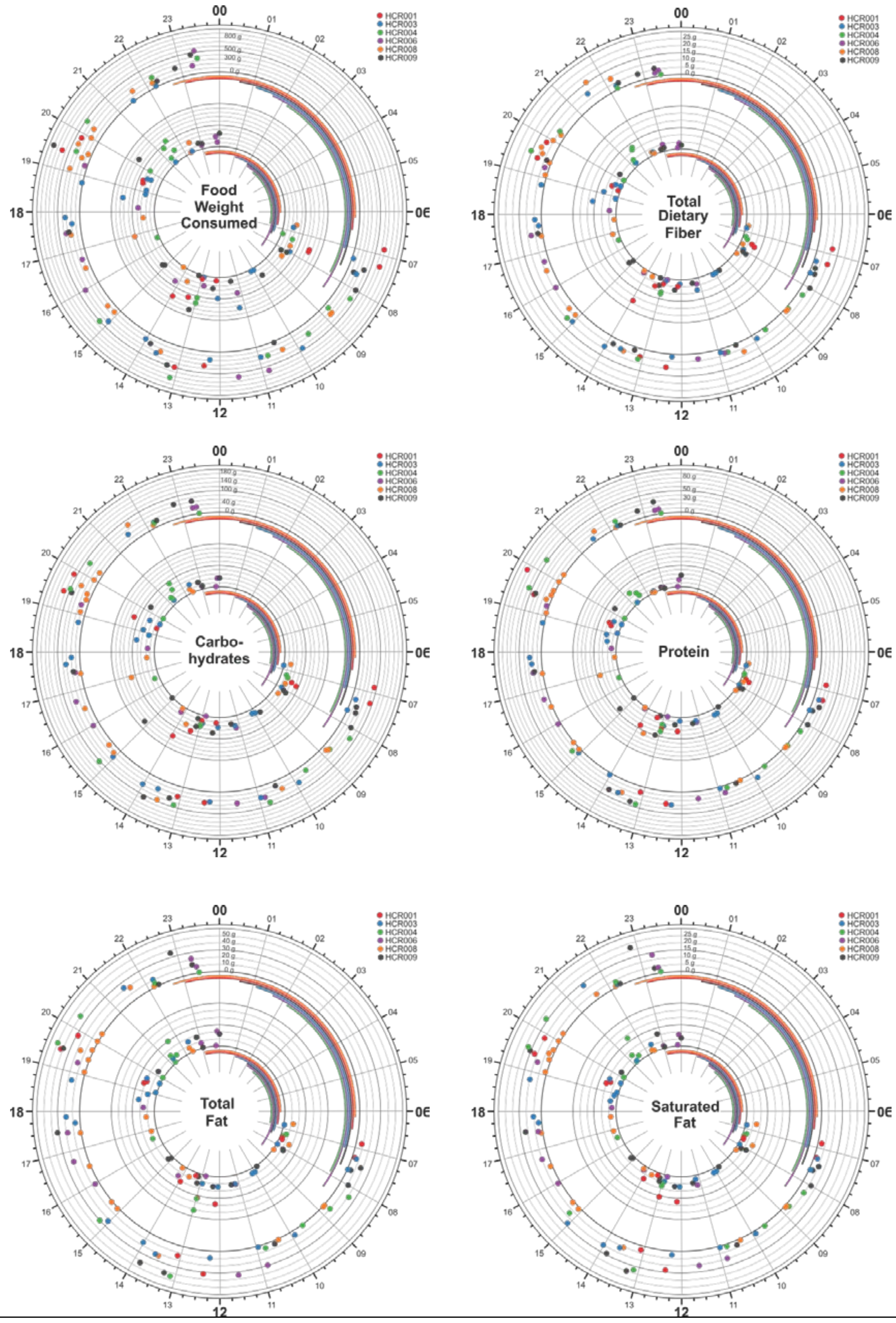

Figure S 3 – Nutritional Assessments - continued

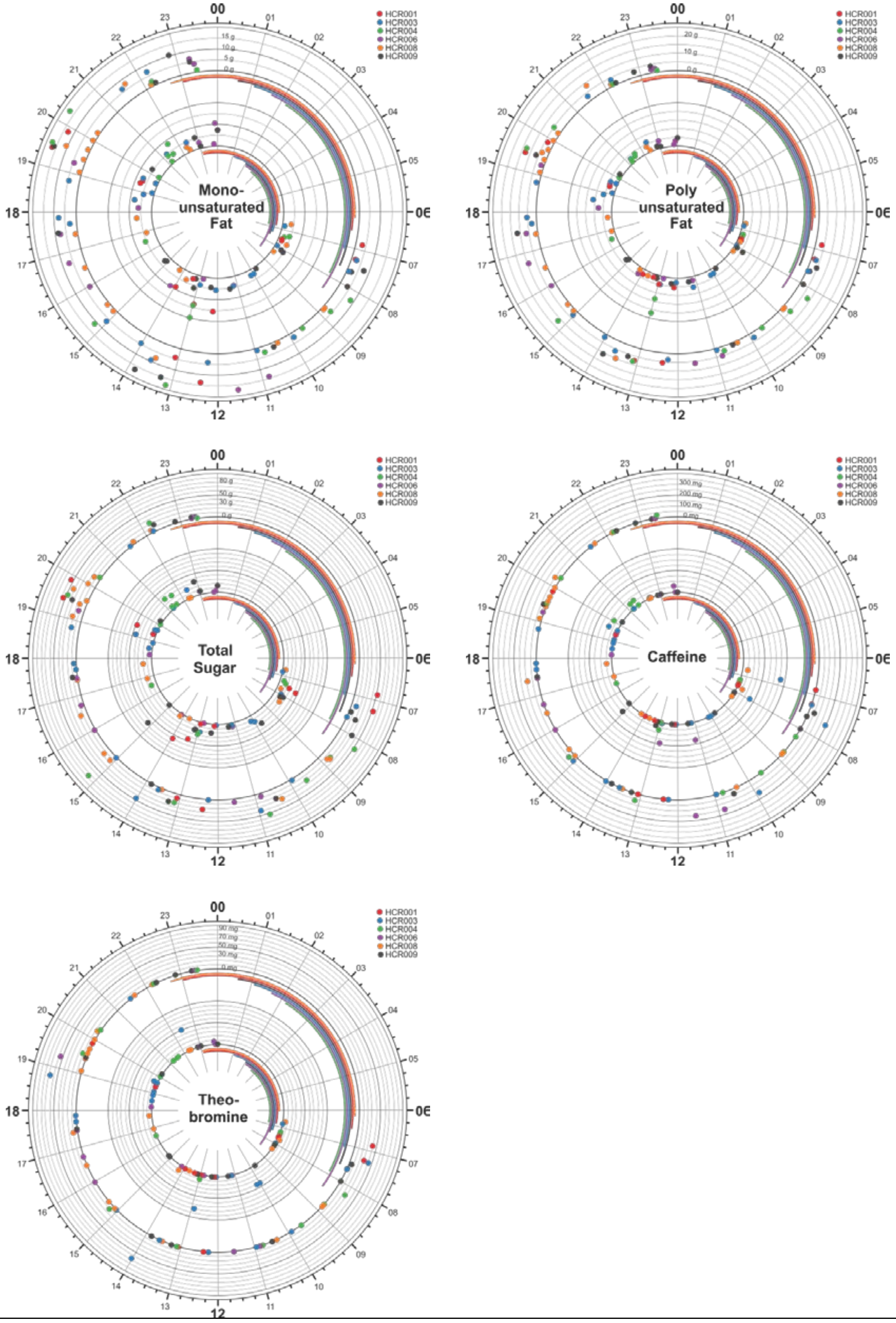

Figure S 4 – Principal Components for Metabolome, Proteome and Microbiome , first 48-hour session

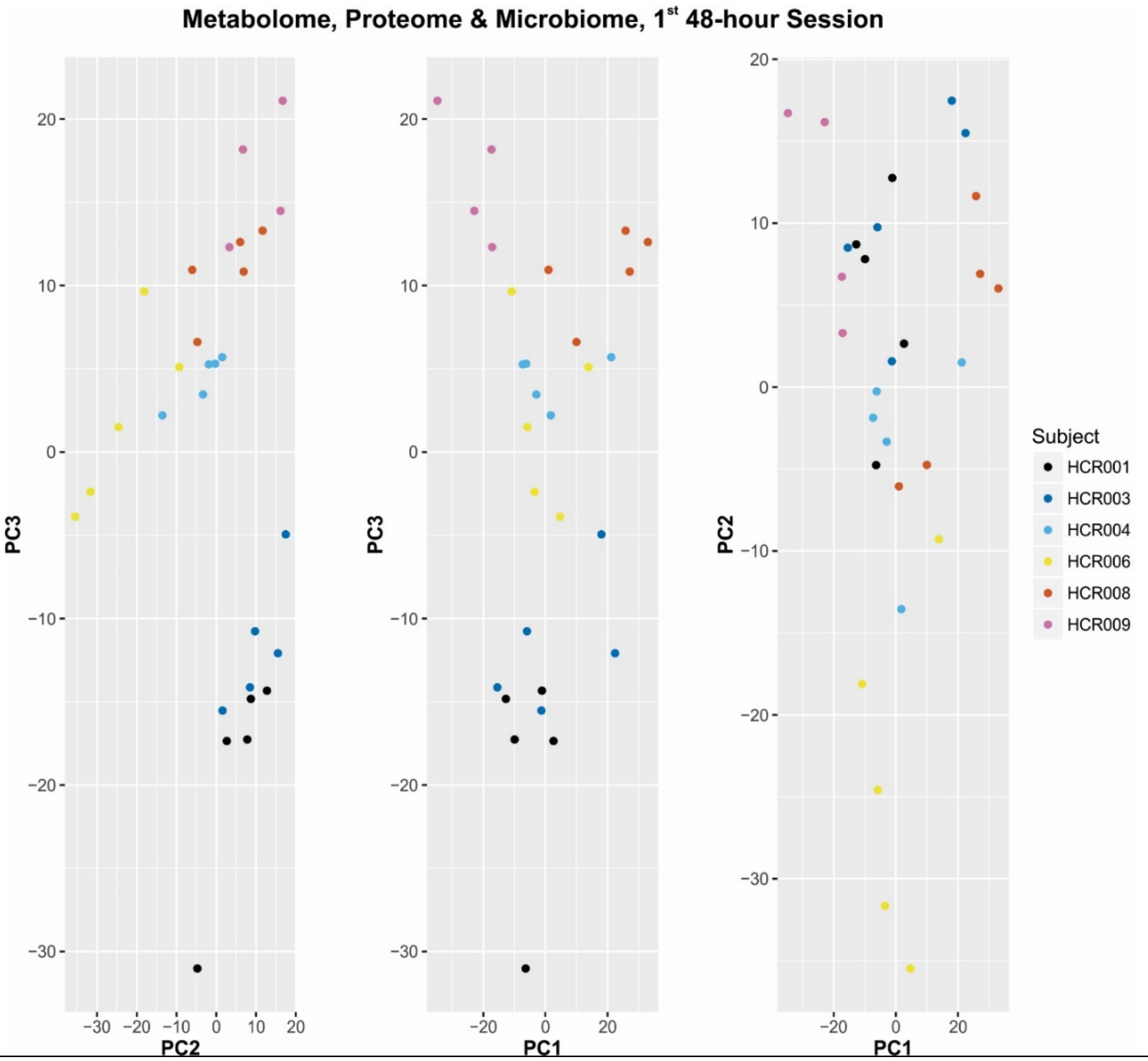

Figure S5a - Networks: Abdominal Neoplasm

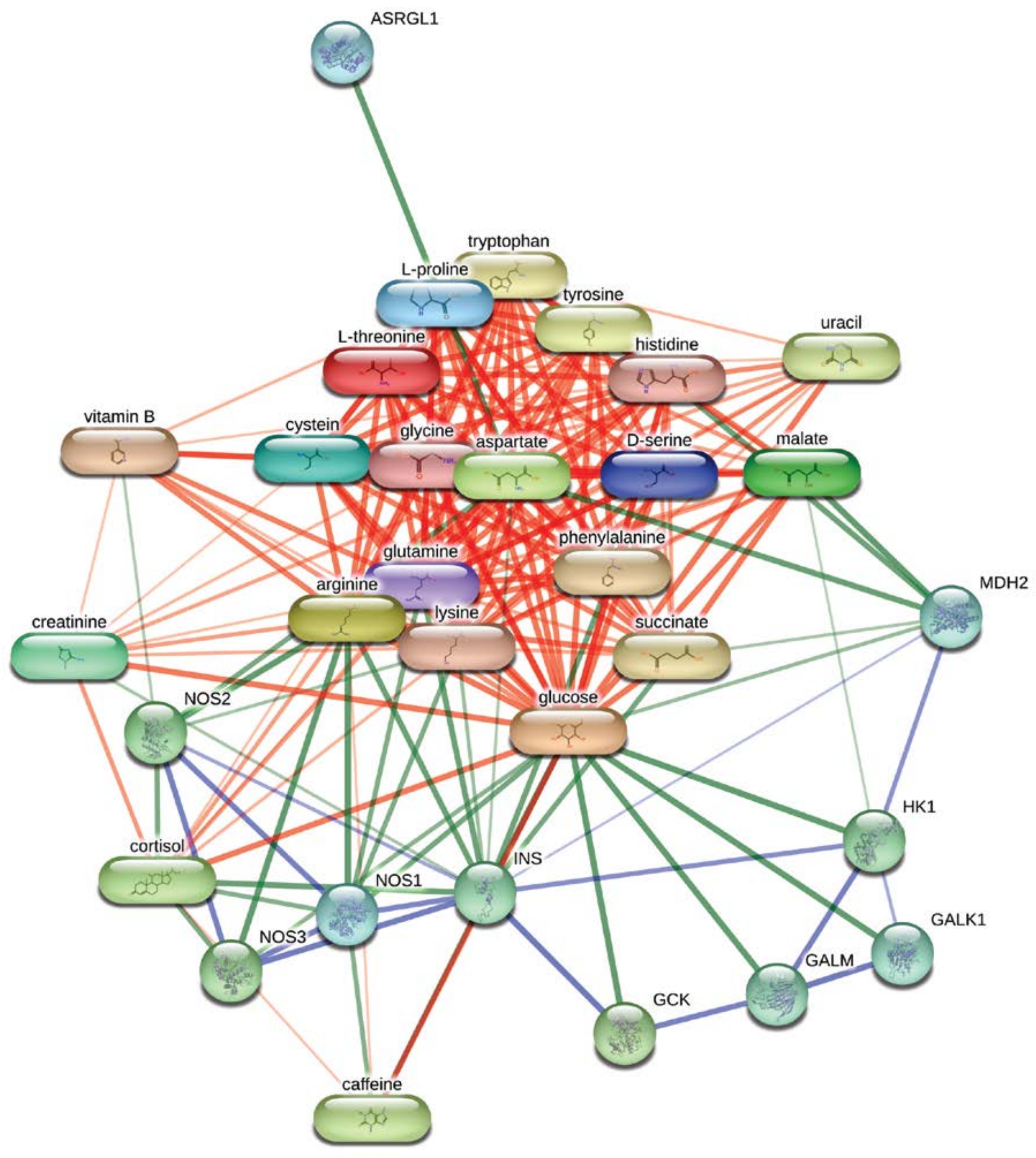

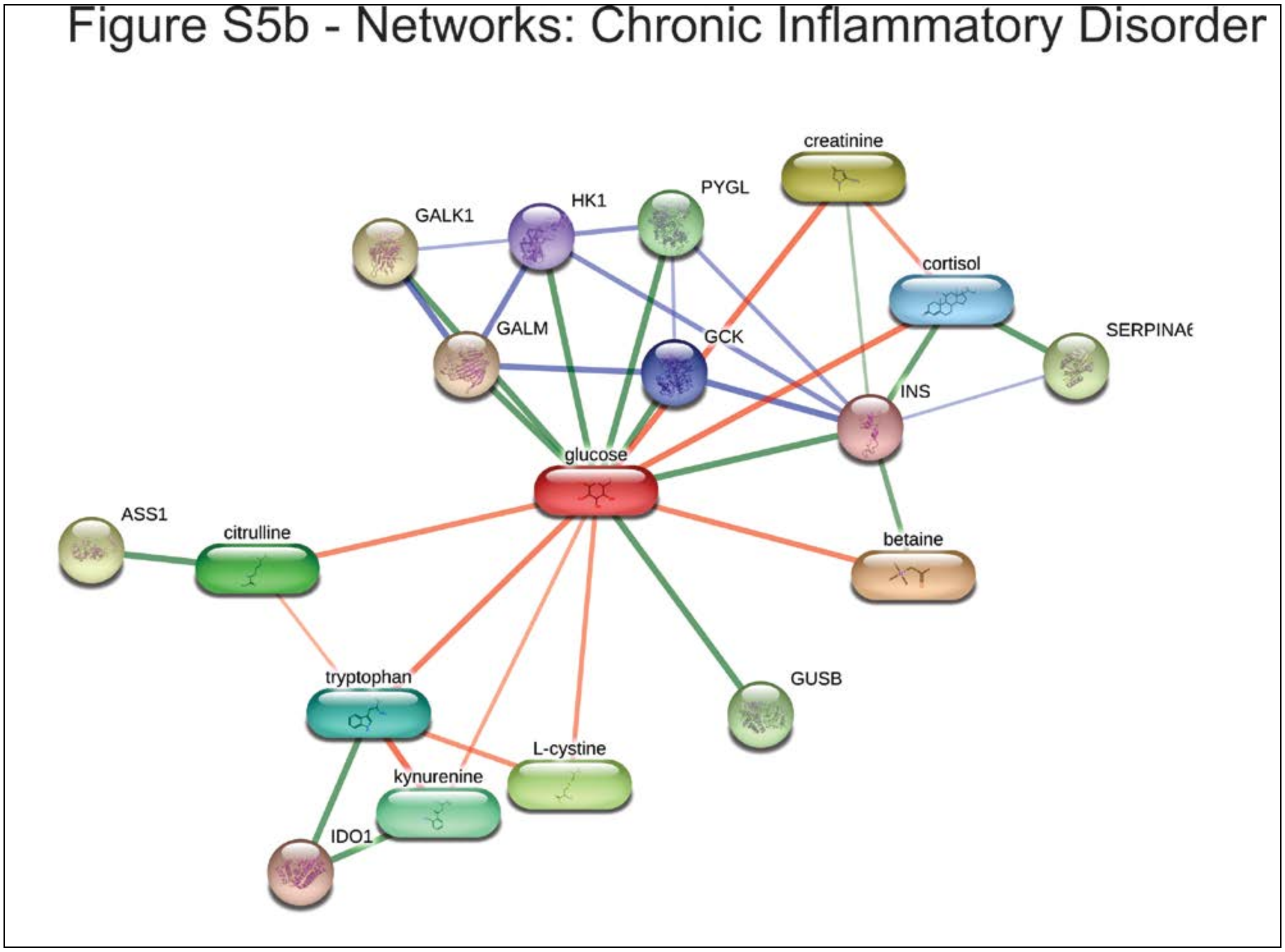
Figure S5b - Networks: Chronic Inflammatory Disorder
creatinine
GALK1
HK1
PYGL
cortisol
GALM
GCK
SERPINA6
INS
glucose
ASS1
citrulline
betaine
tryptophan
GUSB
L-cystine
kynurenine
IDO1

Figure S5c - Networks: Abdominal Neoplasm

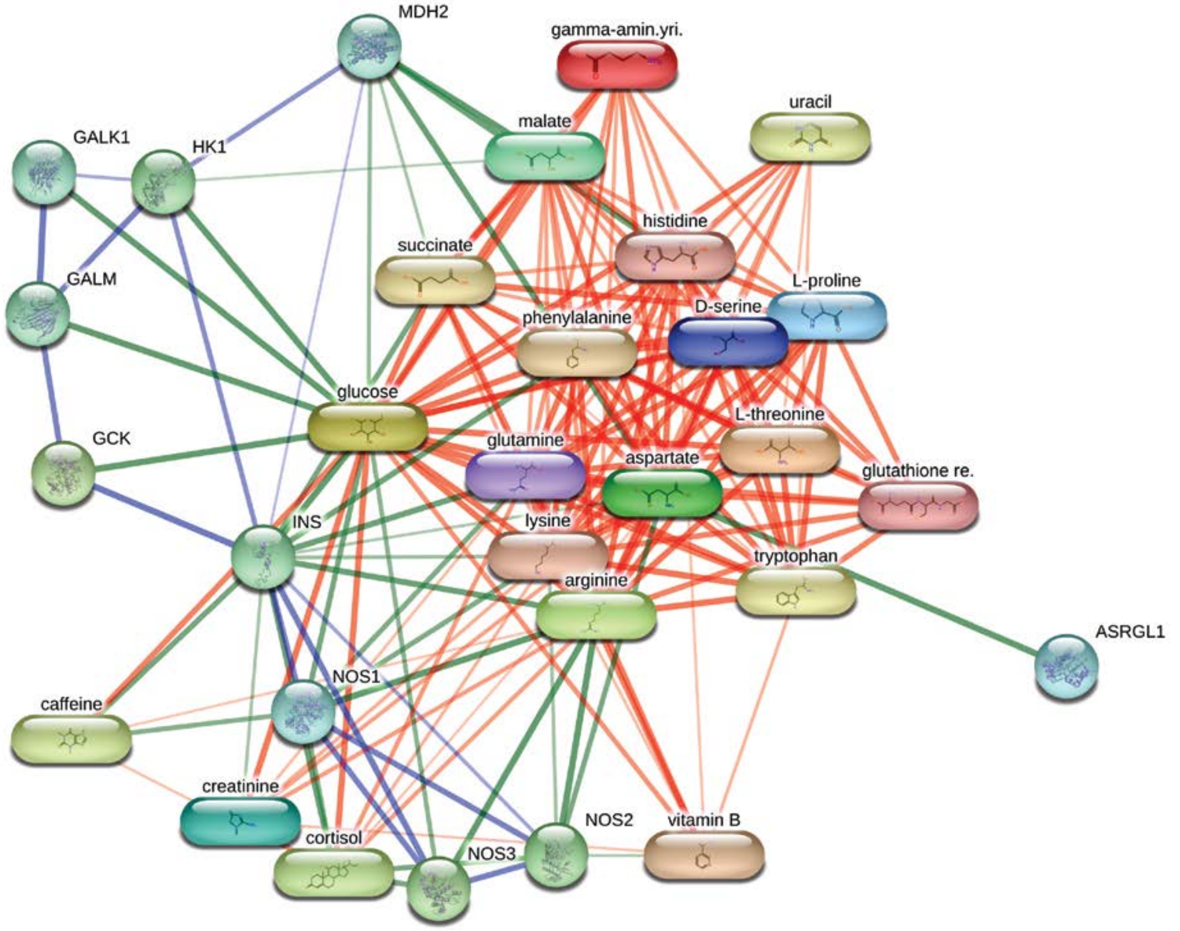

Figure S5d - Networks: Malignant Solid Tumor

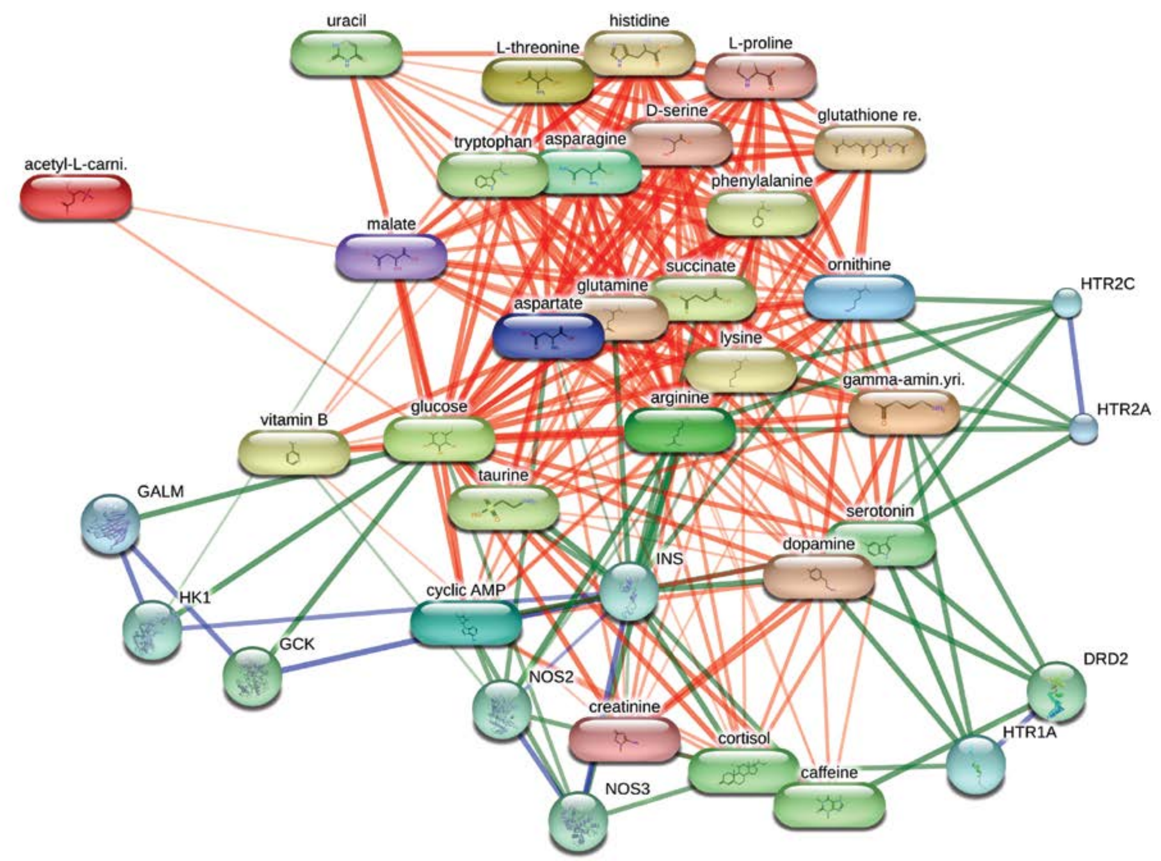

Figure S 6 – Comparison of Subjective and Objective Sleep-Wake Times

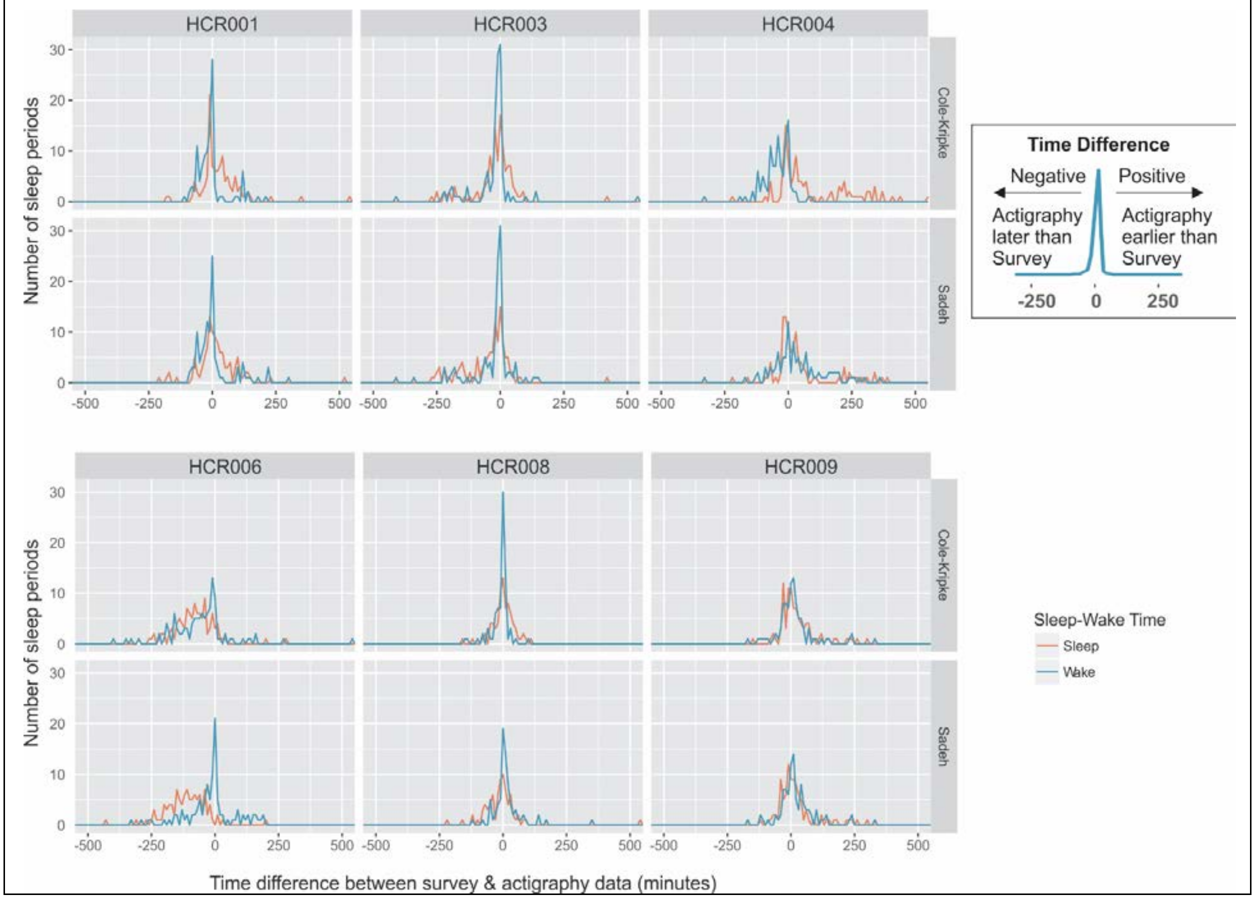

Figure S 7 – Time-of-Day Differences in Microbial Abundances (first study)

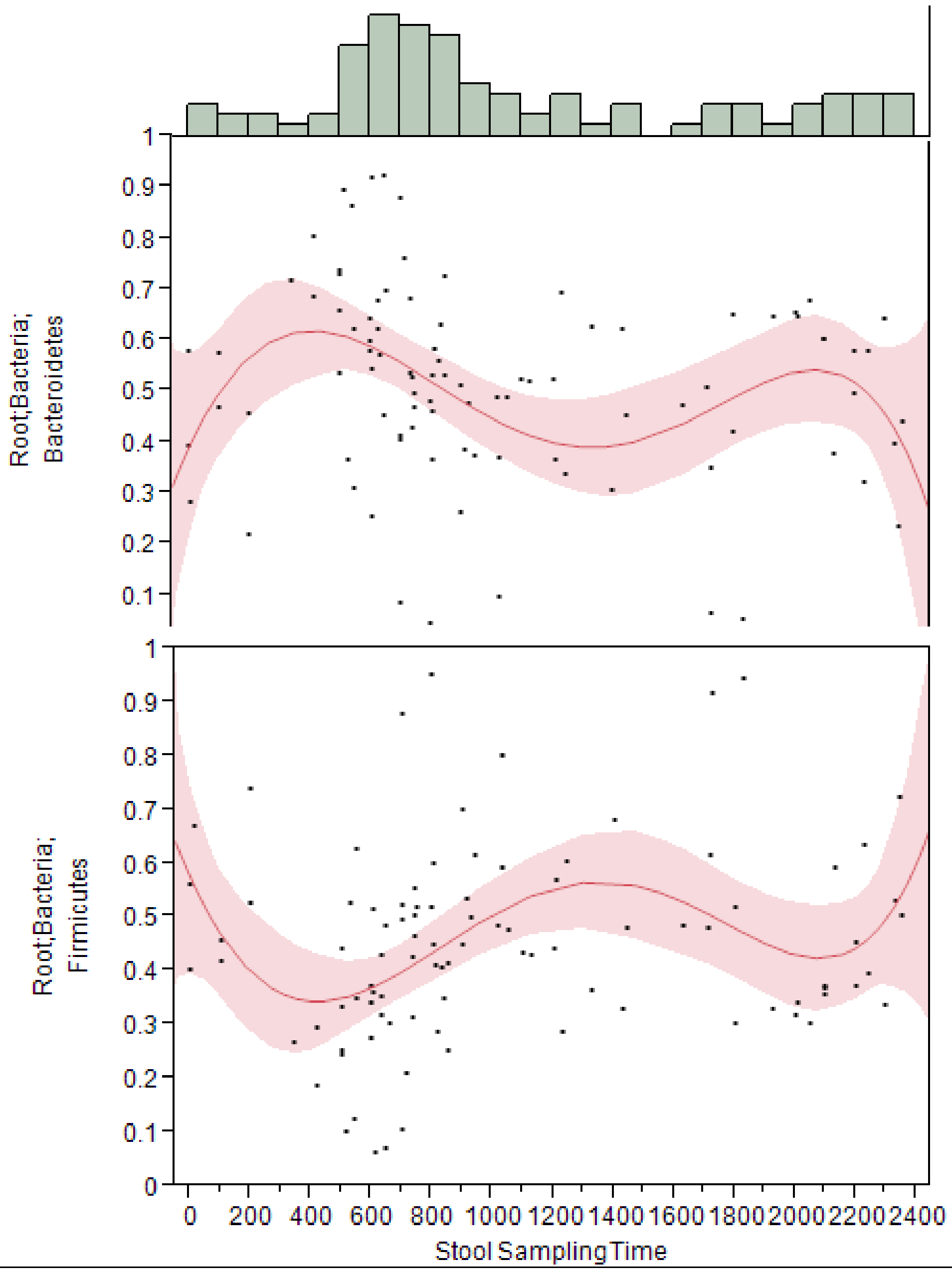

Figure S 8 – Microbiomics UniFrac Distances

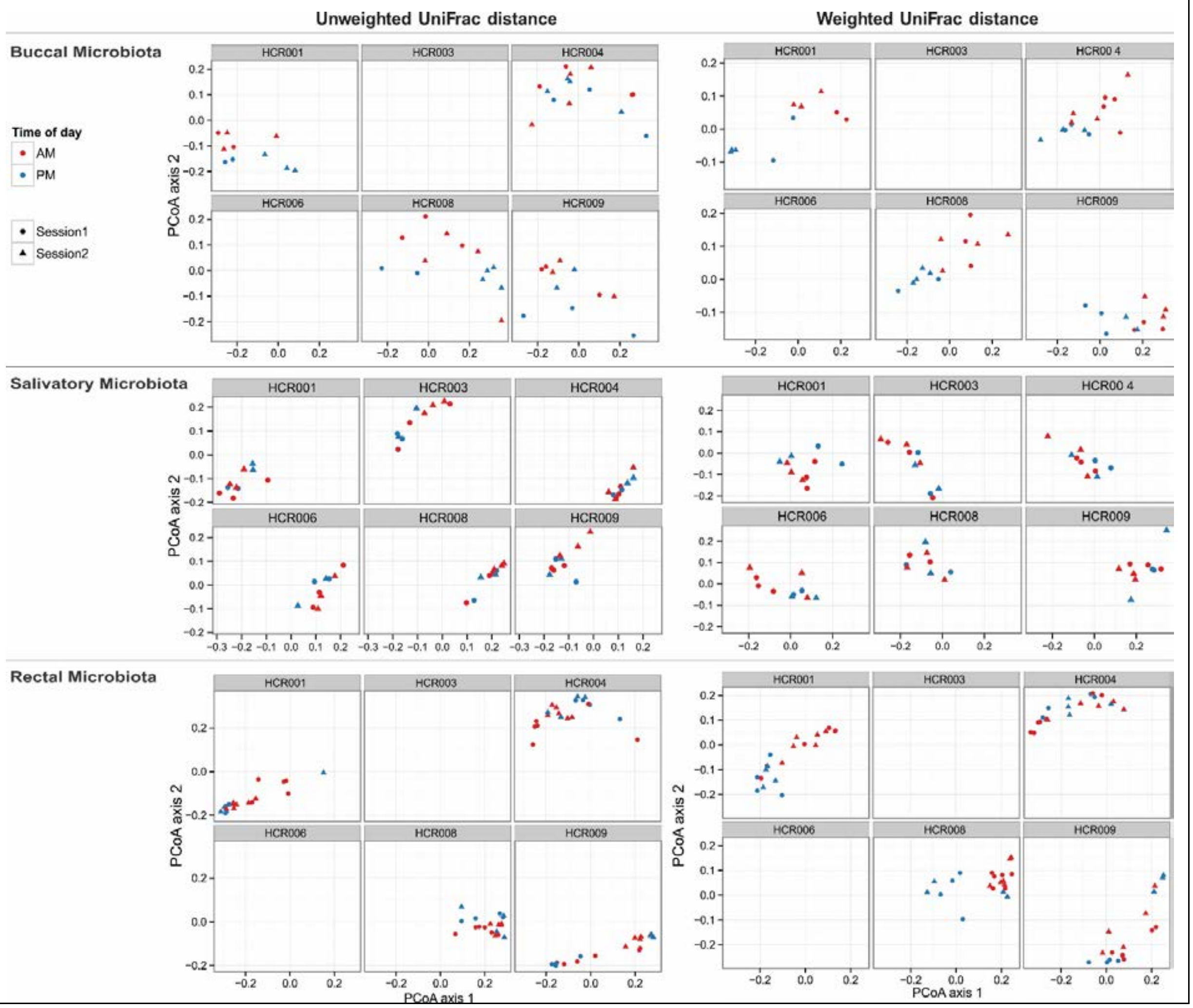

Figure S 9 - Acrophase Alignment

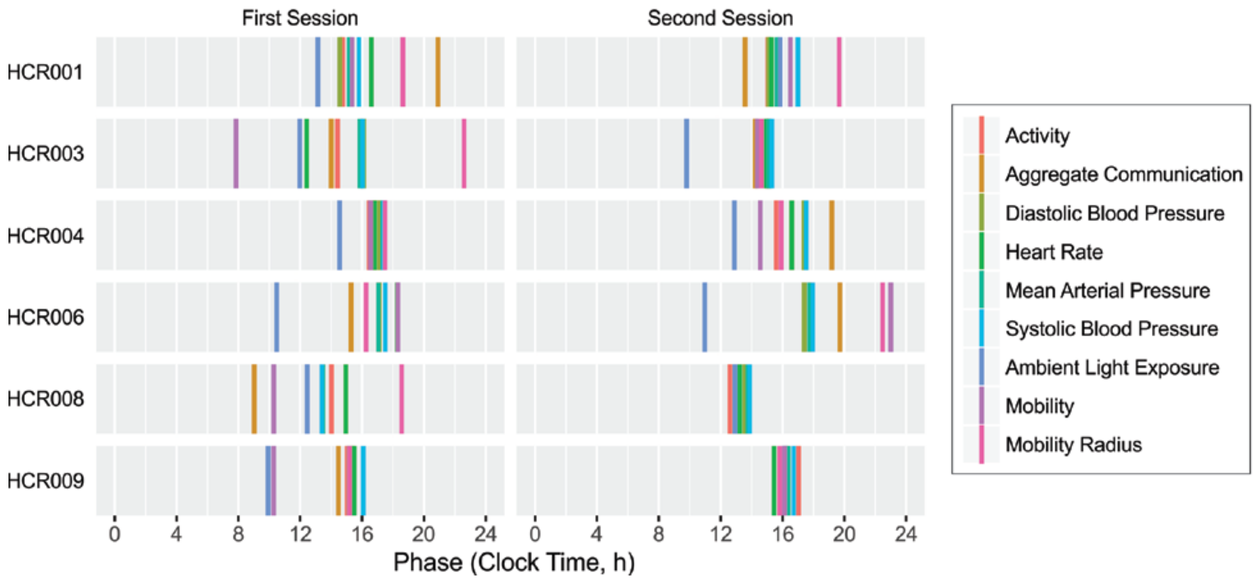

Supplemental Tables

Table S 1 – Demographics

**Subject ID#**	**Gender**	**Age** [years]	**BMI** [kg/m^2]	**Ethnicity**	**Race**
		**at the time of study enrollment**			
**HCR001**	M	32	20.0	Non-Hispanic	Caucasian
**HCR003**	M	26	24.8	Non-Hispanic	Asian
**HCR004**	M	31	25.5	Non-Hispanic	Caucasian
**HCR006**	M	35	26.6	Non-Hispanic	Caucasian
**HCR008**	M	35	30.2	Non-Hispanic	Caucasian
**HCR009**	M	35	23.9	Non-Hispanic	Caucasian
**Mean±SD**		**32.3±3.6**	**25.2±3.4**		

Table S 2 - Adverse Events

ID	Adverse Event	Severity*	Action Taken	Outcome
**HCR001**	Left eye redness	Mild	None	Resolved
**HCR003**	"Scratchy" throat	Mild	None	Resolved
**HCR004**	Blurry vision, left eye	Mild	None	Resolved
**HCR004**	Fever	Mild	None	Resolved
**HCR004**	GI symptoms	Mild	None	Resolved
**HCR006**	Cold symptoms	Mild	None	Resolved
**HCR009**	Headache	Mild	None	Resolved
**HCR009**	Headache	Mild	None	Resolved
**HCR009**	Light headed post blood draw	Mild	None	Resolved

*Criteria for severity: normal, mild, moderate, severe, life-threatening.

Table S 3 - Munich ChronoType Questionnaire (MCTQ)

ID	Chronotype*	Sleep Debt*	Time in bed# [HH:mm]	Sleep latency# [m]	Sleep end# [HH:mm]	Light exposure [h/d]	Time in bed# [HH:mm]	Sleep latency# [m]	Sleep end# [HH:mm]	Light exposure [h/d]
			workdays				work-free days			
**HCR001**	Normal	Up to 30 minutes on free days	23:00	5	07.30 w/o alarm	1	00:00	5	08:00 w/o alarm	1
**HCR003**	Normal	Up to 30 minutes on work days	00:00	30	07:00 w/ alarm	1	01:00	60	09:00 w/o alarm	4
**HCR004**	Slight late	Up to 30 minutes on work days	01:00	15	08:00 w/ alarm	0.75	01:30	15	09:30 w/o alarm	3.5
**HCR006**	Slight late	Not computable	23:30	15	09:30 w/ alarm	2	00:30	15	10:30 w/ alarm	2

**HCR008**	Slight early	Up to 30 minutes on work days	22:00	5	06:00 w/ alarm	0.75	11:00	10	08:00 w/o alarm	2.5
**HCR009**	Normal	Up to 30 minutes on work days	23:50	10	06.30 w/ alarm	1	01:00	10	08:00 w/o alarm	2

*Computed; # Self-reported; 'w/' with; 'w/o' without

ID	Regular Work (Y/N)	Work Start	Work End	Work Duration	Flexibility	Commute to (min)	Commute from (min)	Transport Mode	Shiftwork (Y/N)
HCR001	yes	09:00	17:00	8	very flexible	15	15	walk/bike/etc	no
HCR003	yes	09:00	16:00	7	very flexible	15	15	walk/bike/etc	yes
HCR004	yes	09:00	18:30	9.5	little flexible	15	15	walk/bike/etc	no
HCR006	no	*not answered*	*not answered*	*not answered*	n/a	*not answered*	*not answered*	n/a	no
HCR008	yes	08:00	16:00	8	little flexible	30	30	travel in a vehicle	no
HCR009	yes	08:00	22:00	14	very flexible	15	15	walk/bike/etc	no

Table S 4 - Ambulatory Blood Pressure Monitoring, Session 1 & Session 2

Subject	Blood Pressure [mmHg]	Mean, awake	SD, awake	Mean, asleep	SD, asleep	Mean, delta (awake-asleep)	SD, delta (awake-asleep)	Ratio (Asleep/Awake)	Phenotype
HCR001	SBP	123.4	10.2	105.7	9.6	17.3	5.5	0.86	Dipper
HCR003	SBP	131.6	9.8	111.3	8.8	20.3	1.1	0.85	Dipper
HCR004	SBP	121.8	9.8	97.8	7.5	24.3	2.8	0.80	Dipper
HCR006	SBP	130.4	8.5	109.9	12.6	20.7	4.2	0.84	Dipper
HCR008	SBP	122.2	10.8	98.5	11.6	21.6	0.8	0.81	Dipper
HCR009	SBP	129.4	9.1	110.2	11.6	20.8	4.2	0.85	Dipper
All	***SBP***	***126.1***	***10.6***	***106.8***	***11.6***	***20.7***	***3.9***	***0.85***	***Dipper***
HCR001	DBP	78.3	7.2	58.0	8.7	20.2	4.3		
HCR003	DBP	82.6	7.9	61.9	6.9	21.0	1.5		
HCR004	DBP	74.0	8.2	55.0	6.9	19.1	3.8		
HCR006	DBP	78.6	7.1	59.3	11.3	19.4	4.0		

HCR008	DBP	80.9	11.4	57.9	8.5	25.4	0.6
HCR009	DBP	77.3	9.1	59.4	13.6	21.4	2.5
All	***DBP***	***78.5***	***9.1***	***58.8***	***10.2***	***20.7***	***3.3***
HCR001	MAP	93.0	7.8	75.4	8.5	17.3	4.1
HCR003	MAP	97.6	7.6	78.1	6.9	19.7	1.3
HCR004	MAP	89.5	8.5	70.8	6.6	18.8	3.0
HCR006	MAP	96.1	6.4	77.6	11.2	18.7	4.0
HCR008	MAP	93.7	11.0	70.6	8.8	23.8	0.3
HCR009	MAP	95.1	8.9	77.9	12.8	20.4	2.2
All	***MAP***	***94.0***	***8.9***	***75.8***	***10.1***	***19.4***	***3.2***

Table S 5 - Heart Rate, Session 1 & Session 2

Subject	HR, mean, awake [bpm]	HR, SD, awake [bpm]	HR, mean, asleep [bpm]	HR, SD, asleep [bpm]	HR, mean, delta (awake-asleep) [bpm]	HR, SD, delta (awake-asleep) [bpm]
HCR001	84.6	9.8	67.1	11.0	17.3	2.9
HCR003	66.8	12.1	54.4	7.9	13.1	7.7
HCR004	64.0	11.7	50.1	10.4	13.7	5.8
HCR006	74.3	7.7	63.7	6.9	10.8	4.1
HCR008	75.1	11.3	51.8	5.0	23.4	2.2
HCR009	80.1	11.8	59.1	14.6	24.2	2.7
All	***74.0***	***13.0***	***58.8***	***11.9***	***16.5***	***6.6***

Table S 6 - Social Sensing – Cohort Level

Subject	Parameter	Unit	Sleep Survey	Mean	Min	Max
All	Activity	Counts • min^{-1}	Awake	1904.0	0	55757.7
All	Activity	Counts • min^{-1}	Sleeping	307.2	0	29642.7
All	Ambient light intensity	Lux • min^{-1}	Awake	42.3	0	6500
All	Ambient light intensity	Lux • min^{-1}	Sleeping	2.7	0	5853
All	Aggregate Communication	n	Awake	18.7	0	166
All	Aggregate Communication	n	Sleeping	0.9	0	84
All	Call Count	n	Awake	2.7	0	48
All	Call Count	n	Sleeping	0.1	0	10
All	Call Duration	seconds	Awake	820.4	0	16590
All	Call Duration	seconds	Sleeping	22.0	0	3162
All	Interaction Diversity	n	Awake	7.2	0	71
All	Interaction Diversity	n	Sleeping	0.5	0	29
All	Missed Interaction	n	Awake	0.6	0	9

All	Missed Interaction	n	Sleeping	0.03	0	2
All	Unreturned Calls	n	Awake	0.4	0	8
All	Unreturned Calls	n	Sleeping	0.02	0	2
All	SMS Count	n	Awake	16.0	0	166
All	SMS Count	n	Sleeping	0.8	0	75
All	SMS Length	n (characters)	Awake	868.0	0	14840
All	SMS Length	n (characters)	Sleeping	47.0	0	3089

Table S 7 – Acrophases & Chronotypes

Subject	Measurement Session	Acrophase of Activity [h]	Chronotype according to cutpoints* proposed by Roveda et al. [53]	Chronotype according to MCTQ administered at enrollment
HCR001	Both Sessions	14.9	Neither-type (N)	Normal
HCR003	Both Sessions	14.7	Neither-type (N)	Normal
HCR004	Both Sessions	16.0	Neither-type (N)	Slight late
HCR006	Both Sessions	17.5	Neither-type (N)	Slight late
HCR008	Both Sessions	13.3	Morning-type (M)	Slight early
HCR009	Both Sessions	16.0	Neither-type (N)	Normal

*** Cutpoints defined as: Acrophase of 876 min or 14.6h as morning-type (M); 948 min or 15.8h as neither-type (N); and 1053 min or 17.6h as evening-type (E).**

Supplemental References

1 Danel, T. & Touitou, Y. Alcohol decreases the nocturnal peak of TSH in healthy volunteers. *Psychopharmacology (Berl)* **170**, 213-214, doi:10.1007/s00213-003-1532-9 (2003).

2 Eastman, C. I., Stewart, K. T. & Weed, M. R. Evening alcohol consumption alters the circadian rhythm of body temperature. *Chronobiol Int* **11**, 141-142 (1994).

3 Pedersen, A. K. & FitzGerald, G. A. Cyclooxygenase inhibition, platelet function, and metabolite formation during chronic sulfinpyrazone dosing. *Clin Pharmacol Ther* **37**, 36-42 (1985).

4 Roenneberg, T., Wirz-Justice, A. & Merrow, M. Life between clocks: daily temporal patterns of human chronotypes. *J Biol Rhythms* **18**, 80-90 (2003).

5 Pepperell, J. C. *et al.* Ambulatory blood pressure after therapeutic and subtherapeutic nasal continuous positive airway pressure for obstructive sleep apnoea: a randomised parallel trial. *Lancet* **359**, 204-210, doi:10.1016/s0140-6736(02)07445-7 (2002).

6 Harshfield, G. A., Hwang, C. & Grim, C. E. Circadian variation of blood pressure in blacks: influence of age, gender and activity. *J Hum Hypertens* **4**, 43-47 (1990).

7 Actigraphy white paper, actigraphcorp.com, http://www.actigraphcorp.com/resources/white-papers/ (2008).

8 Troiano, R. P. *et al.* Physical activity in the United States measured by accelerometer. *Med Sci Sports Exerc* **40**, 181-188, doi:10.1249/mss.0b013e31815a51b3 (2008).

9 Cole, R. J., Kripke, D. F., Gruen, W., Mullaney, D. J. & Gillin, J. C. Automatic sleep/wake identification from wrist activity. *Sleep* **15**, 461-469 (1992).

10 Sadeh, A., Sharkey, K. M. & Carskadon, M. A. Activity-based sleep-wake identification: an empirical test of methodological issues. *Sleep* **17**, 201-207 (1994).

11 Tudor-Locke, C., Barreira, T. V., Schuna, J. M., Jr., Mire, E. F. & Katzmarzyk, P. T. Fully automated waist-worn accelerometer algorithm for detecting children's sleep-period time separate from 24-h physical activity or sedentary behaviors. *Appl Physiol Nutr Metab* **39**, 53-57, doi:10.1139/apnm-2013-0173 (2014).

12 Skarke, C. *et al.* Bioactive products formed in humans from fish oils. *J Lipid Res* **56**, 1808-1820, doi:10.1194/jlr.M060392 (2015).

13 Murphy, R. C. Specialized pro-resolving mediators: do they circulate in plasma? *J Lipid Res* **56**, 1641-1642, doi:10.1194/jlr.C062356 (2015).

14 McKenna, P. *et al.* The macaque gut microbiome in health, lentiviral infection, and chronic enterocolitis. *PLoS Pathog* **4**, e20, doi:07-PLPA-RA-0170 (2008).

15 Wu, G. D. *et al.* Sampling and pyrosequencing methods for characterizing bacterial communities in the human gut using 16S sequence tags. *BMC Microbiol* **10**, 206, doi:10.1186/1471-2180-10-206 (2010).

16 Caporaso, J. G. *et al.* QIIME allows analysis of high-throughput community sequencing data. *Nat Methods* **7**, 335-336, doi:10.1038/nmeth.f.303 (2010).

17 Edgar, R. C. Search and clustering orders of magnitude faster than BLAST. *Bioinformatics* **26**, 2460-2461, doi:10.1093/bioinformatics/btq461 (2010).

18 Caporaso, J. G. *et al.* PyNAST: a flexible tool for aligning sequences to a template alignment. *Bioinformatics* **26**, 266-267, doi:10.1093/bioinformatics/btp636 (2010).

19 Price, M. N., Dehal, P. S. & Arkin, A. P. FastTree 2--approximately maximum-likelihood trees for large alignments. *PLoS One* **5**, e9490, doi:10.1371/journal.pone.0009490 (2010).

20 McDonald, D. *et al.* An improved Greengenes taxonomy with explicit ranks for ecological and evolutionary analyses of bacteria and archaea. *ISME J* **6**, 610-618, doi:10.1038/ismej.2011.139 (2012).

21 Yuan, M., Breitkopf, S. B., Yang, X. & Asara, J. M. A positive/negative ion-switching, targeted mass spectrometry-based metabolomics platform for bodily fluids, cells, and fresh and fixed tissue. *Nat Protoc* **7**, 872-881, doi:10.1038/nprot.2012.024 (2012).
22 Gold, L. *et al.* Aptamer-based multiplexed proteomic technology for biomarker discovery. *PLoS One* **5**, e15004, doi:10.1371/journal.pone.0015004 (2010).
23 SomaLogic. SOMAscan Technical White Paper. © 2015 SomaLogic, Inc. • SSM-002, Rev. 3 • Effective: 9/21/2015 • DCN 15-310 (2015).
24 Martin, C. K. *et al.* Validity of the Remote Food Photography Method (RFPM) for estimating energy and nutrient intake in near real-time. *Obesity (Silver Spring)* **20**, 891-899, doi:10.1038/oby.2011.344 (2012).
25 Martin, C. K. *et al.* A novel method to remotely measure food intake of free-living individuals in real time: the remote food photography method. *Br J Nutr* **101**, 446-456, doi:10.1017/S0007114508027438 (2009).
26 Shiffman, S., Stone, A. A. & Hufford, M. R. Ecological momentary assessment. *Annu Rev Clin Psychol* **4**, 1-32 (2008).
27 Ahuja, J. K. C. *et al.* USDA Food and Nutrient Database for Dietary Studies, 5.0, 2012. *U.S. Department of Agriculture, Agricultural Research Service, Food Surveys Research Group, Beltsville, MD* (2012).
28 Williamson, D. A. *et al.* Digital photography: a new method for estimating food intake in cafeteria settings. *Eat Weight Disord* **9**, 24-28 (2004).
29 Williamson, D. A. *et al.* Comparison of digital photography to weighed and visual estimation of portion sizes. *J Am Diet Assoc* **103**, 1139-1145, doi:10.1053/jada.2003.50567 (2003).
30 Feskanich, D., Sielaff, B. H., Chong, K. & Buzzard, I. M. Computerized collection and analysis of dietary intake information. *Comput Methods Programs Biomed* **30**, 47-57 (1989).
31 Johnson, R. K., Driscoll, P. & Goran, M. I. Comparison of multiple-pass 24-hour recall estimates of energy intake with total energy expenditure determined by the doubly labeled water method in young children. *J Am Diet Assoc* **96**, 1140-1144, doi:S0002-8223(96)00293-3 (1996).
32 Harnack, L. *et al.* A computer-based approach for assessing dietary supplement use in conjunction with dietary recalls. *J Food Compost Anal* **21**, S78-S82, doi:10.1016/j.jfca.2007.05.004 (2008).
33 Sievert, Y. A., Schakel, S. F. & Buzzard, I. M. Maintenance of a nutrient database for clinical trials. *Control Clin Trials* **10**, 416-425 (1989).
34 Schakel, S. F., Sievert, Y. A. & Buzzard, I. M. Sources of data for developing and maintaining a nutrient database. *J Am Diet Assoc* **88**, 1268-1271 (1988).
35 Schakel, S., Buzzard, I. & Gebhardt, S. Procedures for estimating nutrient values for food composition databases. . *J Food Comp Anal* 102-114 (1997).
36 Harris, P. A. *et al.* Research electronic data capture (REDCap)--a metadata-driven methodology and workflow process for providing translational research informatics support. *J Biomed Inform* **42**, 377-381, doi:10.1016/j.jbi.2008.08.010 (2009).
37 Anderson, M. J. A new method for non-parametric multivariate analysis of variance. *Austral Ecology* **26**, 32-46 (2001).
38 Lozupone, C. & Knight, R. UniFrac: a new phylogenetic method for comparing microbial communities. *Appl Environ Microbiol* **71**, 8228-8235, doi:10.1128/AEM.71.12.8228-8235.2005 (2005).

39 Lozupone, C. A., Hamady, M., Kelley, S. T. & Knight, R. Quantitative and qualitative beta diversity measures lead to different insights into factors that structure microbial communities. *Appl Environ Microbiol* **73**, 1576-1585, doi:10.1128/AEM.01996-06 (2007).

40 Price, T. S., Baggs, J. E., Curtis, A. M., Fitzgerald, G. A. & Hogenesch, J. B. WAVECLOCK: wavelet analysis of circadian oscillation. *Bioinformatics* **24**, 2794-2795, doi:10.1093/bioinformatics/btn521 (2008).

41 Morris, J. M. & Peravali, R. Minimum-bandwidth discrete-time wavelets. *Signal Processing* **76**, 181-193 (1999).

42 Cornelissen, G. Cosinor-based rhythmometry. *Theor Biol Med Model* **11**, 16, doi:10.1186/1742-4682-11-16 (2014).

43 Refinetti, R., Lissen, G. C. & Halberg, F. Procedures for numerical analysis of circadian rhythms. *Biol Rhythm Res* **38**, 275-325, doi:10.1080/09291010600903692 (2007).

44 Neter, J. & Wassermann, W. Applied Linear Statistical Models. *WCB/McGraw-Hill* **4. Edition**, 121 (1996).

45 Fagard, R. H. *et al.* Night-day blood pressure ratio and dipping pattern as predictors of death and cardiovascular events in hypertension. *J Hum Hypertens* **23**, 645-653, doi:10.1038/jhh.2009.9 (2009).

46 Muxfeldt, E. S., Cardoso, C. R. & Salles, G. F. Prognostic value of nocturnal blood pressure reduction in resistant hypertension. *Arch Intern Med* **169**, 874-880, doi:10.1001/archinternmed.2009.68 (2009).

47 Santos-Lozano, A. *et al.* Actigraph GT3X: validation and determination of physical activity intensity cut points. *Int J Sports Med* **34**, 975-982, doi:10.1055/s-0033-1337945 (2013).

48 Paquet, J., Kawinska, A. & Carrier, J. Wake detection capacity of actigraphy during sleep. *Sleep* **30**, 1362-1369 (2007).

49 Tandon, P. S., Saelens, B. E., Zhou, C., Kerr, J. & Christakis, D. A. Indoor versus outdoor time in preschoolers at child care. *Am J Prev Med* **44**, 85-88, doi:10.1016/j.amepre.2012.09.052 (2013).

50 Bunning, E. & Moser, I. Interference of moonlight with the photoperiodic measurement of time by plants, and their adaptive reaction. *Proc Natl Acad Sci U S A* **62**, 1018-1022 (1969).

51 Gill, S. & Panda, S. A Smartphone App Reveals Erratic Diurnal Eating Patterns in Humans that Can Be Modulated for Health Benefits. *Cell Metab* **22**, 789-798, doi:10.1016/j.cmet.2015.09.005 (2015).

52 Champagne, C. M. *et al.* Energy intake and energy expenditure: a controlled study comparing dietitians and non-dietitians. *J Am Diet Assoc* **102**, 1428-1432 (2002).

53 Roveda, E. *et al.* Predicting the actigraphy-based acrophase using the Morningness-Eveningness Questionnaire (MEQ) in college students of North Italy. *Chronobiol Int* **34**, 551-562, doi:10.1080/07420528.2016.1276928 (2017).
